# *De Novo* Venom Gland Transcriptome Assembly and Characterization for *Calloselasma rhodostoma* (Kuhl, 1824), the Malayan Pit Viper from Malaysia: Unravelling Toxin Gene Diversity in a Medically Important Basal Crotaline

**DOI:** 10.3390/toxins15050315

**Published:** 2023-04-29

**Authors:** Choo Hock Tan, Kae Yi Tan, Tzu Shan Ng, Nget Hong Tan, Ho Phin Chong

**Affiliations:** 1 Department of Pharmacology, Faculty of Medicine, Universiti Malaya, Kuala Lumpur 50603, Malaysia; chong.hophin@um.edu.my; 2 Department of Molecular Medicine, Faculty of Medicine, Universiti Malaya, Kuala Lumpur 50603, Malaysia; kytan_kae@um.edu.my (K.Y.T.); tzushang@gmail.com (T.S.N.); tanngethong@yahoo.com.sg (N.H.T.)

**Keywords:** *Calloselasma rhodostoma*, venom gland, transcriptome, toxins diversity

## Abstract

In Southeast Asia, the Malayan Pit Viper (*Calloselasma rhodostoma*) is a venomous snake species of medical importance and bioprospecting potential. To unveil the diversity of its toxin genes, this study *de novo* assembled and analyzed the venom gland transcriptome of *C. rhodostoma* from Malaysia. The expression of toxin genes dominates the gland transcriptome by 53.78% of total transcript abundance (based on overall FPKM, Fragments Per Kilobase Million), in which 92 non-redundant transcripts belonging to 16 toxin families were identified. Snake venom metalloproteinase (SVMP, PI > PII > PIII) is the most dominant family (37.84% of all toxin FPKM), followed by phospholipase A_2_ (29.02%), bradykinin/angiotensin-converting enzyme inhibitor-C-type natriuretic peptide (16.30%), C-type lectin (CTL, 10.01%), snake venom serine protease (SVSP, 2.81%), L-amino acid oxidase (2.25%), and others (1.78%). The expressions of SVMP, CTL, and SVSP correlate with hemorrhagic, anti-platelet, and coagulopathic effects in envenoming. The SVMP metalloproteinase domains encode hemorrhagins (kistomin and rhodostoxin), while disintegrin (rhodostomin from P-II) acts by inhibiting platelet aggregation. CTL gene homologues uncovered include rhodocytin (platelet aggregators) and rhodocetin (platelet inhibitors), which contribute to thrombocytopenia and platelet dysfunction. The major SVSP is a thrombin-like enzyme (an ancrod homolog) responsible for defibrination in consumptive coagulopathy. The findings provide insight into the venom complexity of *C. rhodostoma* and the pathophysiology of envenoming.

## 1. Introduction

Venomous snakes of the Elapidae and Viperidae families are medically important snakes responsible for most snakebite envenoming cases [1]. Between the two clades, the elapids are relatively younger (less than 40 Mya old) [2], while the viperids first radiated more than 40–50 million years ago in the Old World, followed by invasion and diversification in the New World [3]. To date, viperids comprise more than 300 species grouped under the three subfamilies of Azemiopinae (Feas vipers), Viperinae (true vipers), and Crotalinae (pit vipers) [3]. The crotalines (pit vipers) are arguably the most speciose (with at least 280 species), extensively distributed on all continents except Antarctica, Australia, islands far from continents such as Hawaii and Madagascar, and various isolated islets [4]. Among the Old World crotalines, a basal clade of pit vipers consisting of the species-poor genera *Calloselasma*, *Deinagkistrodon*, *Garthius*, *Hypnale,* and *Tropidolaemus* was recognized [3]. The snakes of *Calloselasma*, *Deinagkistrodon,* and *Hypnale* are particularly well known for causing severe snakebite envenoming in regions where they are prevalent, i.e., Southeast Asia, East Asia, and South Asia, respectively [5,6,7]. These species are designated by the World Health Organization (WHO) as Category 1 medically important venomous snakes, corresponding to their wide distribution and association with high mortality and morbidity [8].

**Figure 1:**
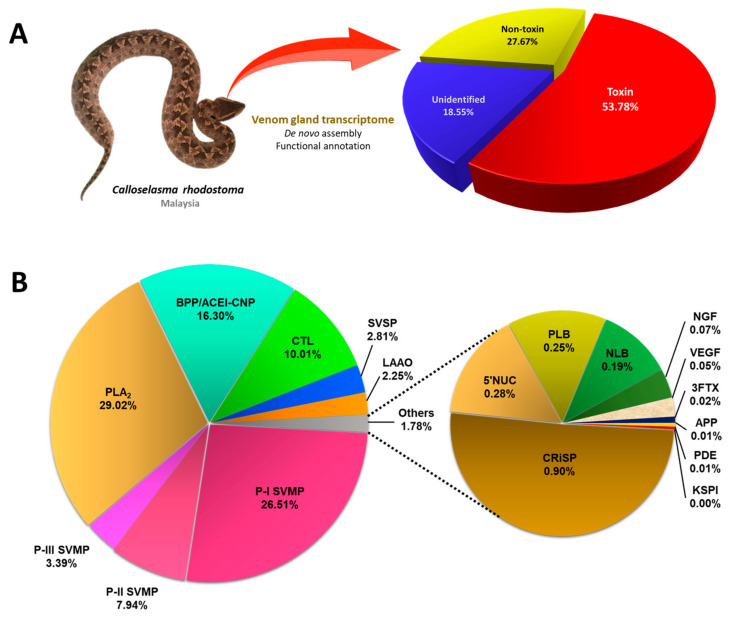
**The transcriptomic profile of *Calloselasma rhodostoma* venom gland.** (**A**) Classification of transcripts into toxins, non-toxins, and unidentified genes derived from *de novo* transcriptome assembly of the venom gland of Malayan Pit Viper (inset). (**B**) Profiling of toxin transcripts into 16 families of genes coding for various venom proteins. Percentages indicate the relative abundances of transcripts based on FPKM. Abbreviations: PLA_2_, phospholipase A_2_; SVMP, snake venom metalloproteinase; BPP/ACEI-CNP, bradykinin-potentiating peptide/angiotensin-converting enzyme inhibitor-C-type natriuretic peptide; CTL, snake C-type lectin; SVSP, snake venom serine proteinase; LAAO, L-amino acid oxidase; NGF, nerve growth factor; CRiSP, cysteine-rich secretory protein; PLB, phospholipase-B; VEGF, vascular endothelial growth factor; 5′NT, 5′-nucleotidase; 3FTX, three-finger toxin; KSPI, Kunitz-type serine proteinase inhibitor; APP, aminopeptidase A; PDE, phosphodiesterase; NLB, nucleobindin.

In Southeast Asia, the genus *Calloselasma* is monotypic and represented by only one species, *Calloselasma rhodostoma*, whose type locality is Java [9]. Commonly known as the Malayan Pit Viper, this crotaline is found in the peninsula of Malaysia (northern west region), most parts of Indochina (Thailand, Laos, Cambodia, and Vietnam), and in some islands of Indonesia (Java, Karimunjawa, and Kangean) [4,10]. Moreover, there were descriptions of sightings of this species in the middle part of Peninsular Malaysia and part of Borneo Island (in Kalimantan, where the snakes might have been introduced for pest control in plantations)—Apparently, these are non-native and invasive populations with broad impacts on the local biodiversity, economics, and public health. On average, an adult Malayan Pit Viper measures about 75 cm long, with females being slightly longer than males [11]. The body of the Malayan Pit Viper is stout, dorsally reddish, grayish, or pale brown, flanked by two alternating series of large, dark brown, black-edged triangular blotches (see graphical abstract and Figure 1). The species is unique among many Asiatic viperids with its large crown scales and smooth dorsal scales. It has a reputation for being bad-tempered and quick to strike when disturbed, but it also tends to stay in the same spot even hours after biting a person. This sedentary habit most likely earned it the local Malay name “ular kapak bodoh”, which is literally translated as “stupid viper” [12]. 

Clinically, envenoming caused by *C. rhodostoma* is highly toxic and fatal. *C. rhodostoma* envenoming results in local tissue damage and systemic hemotoxicity characterized by defibrination coagulopathy and thrombocytopenia [13,14,15]. The hemotoxic envenoming manifests as petechiae, epistaxis, hematuria, hemoptysis, and consumptive coagulopathy, which may lead to complications such as intracranial hemorrhage with deadly sequelae [8,16]. Earlier, a number of proteomic studies provided information on *C. rhodostoma* venom composition at varying depths [17,18,19,20,21]. A more thorough, multi-step, quantitative proteomic study unveiled at least 96 distinct proteins belonging to 11 families in the *C. rhodostoma* venom of Malayan origin [18]. The venom consists of mainly snake venom metalloproteinases (SVMP, 41.17% of total venom proteins), comprising the P-I (kistomin, 20.4%) and P-II (rhodostoxin, 19.8%) classes of SVMP. Other toxins identified were protein families of C-type lectin (snaclec, 26.3%), snake venom serine protease (SVSP, 14.9%), L-amino acid oxidase (7.0%), phospholipase A_2_ (4.4%), cysteine-rich secretory protein (2.5%), and some minor proteins totaling 2.6%. In brief, the pathophysiology of *C. rhodostoma* systemic envenoming involves a constellation of hemotoxic effects caused by multiple toxins in the venom: SVSP ancrod causes venom-induced consumptive coagulopathy, aggravated by thrombocytopenia secondary to platelet aggregation induced by snaclec rhodocytin, while P-II SVMP rhodostoxin causes hemorrhage in various tissues, exacerbated by P-I SVMP kistomin and snaclec rhodocetin that inhibit platelet plug formation. Local bleeding, inflammation and tissue necrosis at the bite site are likely caused by hemorrhagin SVMP, cytotoxic PLA_2_ and LAAO in the venom [22]. 

The proteomic findings of *C. rhodostoma* venom will be useful information for antivenom design and for bioprospecting of toxins in drug discovery [23]. However, a comprehensive gene profile that reflects the venom complexity of this species remains unavailable. We anticipate that a *de novo* assembly of its venom gland transcriptome will shed light on the diversity of venom genes expressed by *C. rhodostoma*. The findings will provide insights into the pathophysiology of *C. rhodostoma* envenoming, allowing practitioners to devise effective monitoring and treatment plans tailored to individual toxins in the venom. The uncovering of specific toxin gene sequences shall also contribute to the knowledge database of snake venoms. This will enable the comparison of toxin genes between species, uncovering their conserved and divergent antigenicity, thereby facilitating the development of a pan-regional antivenom that targets specific toxins from various species. Hence, by applying the high-throughput next-generation sequencing approach, this study is the first to report the *de novo* transcriptome assembly, functional annotation, and expression profiling for the venom gland of *C. rhodostoma* from Malaysia. The diversity of toxin genes in this species was further elucidated on the basis of its implications for snakebite envenoming and potential biomedical applications. 

## 2. Results and Discussion

### 2.1. De Novo Transcriptome Assembly, Transcript Categorization, and Expression

*De novo* sequencing by Illumina HiSeq™ 2000 yielded a total of 46,636,384 clean reads, from which 146,916 contigs (N50 = 632) were assembled through Trinity (Q20 percentage = 97.17%) (Table 1). These contigs were connected, resulting in 74,445 transcripts (N50 = 1636), validated in the NCBI non-redundant (NR) protein database through BLASTx (e-value < 10^−5^) (Supplementary File S1). The low-frequency transcripts (FPKM < 1) were filtered as non-expressing, and the remaining assemblies of 59,348 transcripts were further grouped into “unidentified”, “non-toxin,” and “toxin” categories accordingly. The “toxin” group composed 53.78% of the total transcript expression (FPKM), comprising 97 putative or classical toxin transcripts. The “non-toxin” and “unidentified” groups constituted 27.67% (20,962 transcripts) and 18.55% (38,289 transcripts), respectively, of the overall transcriptome (Table 1; Figure 1). The total expression level of toxin transcripts in *C. rhodostoma* venom gland (~54%) was high and comparable to pit vipers reported previously, for instance, *Bothrops atrox* (~59%) [24], *Deinagkistrodon acutus* (~40%) [25], and *Gloydius intermedius* (~61%) [26]. The high expressions of toxin transcripts were accompanied by a small subset of genes (97 out of 59,348), giving rise to a high redundancy of toxin genes (4549.90 FPKM/transcript). In contrast, the groups of “non-toxin” (10.39 FPKM/transcript) and “unidentified” (3.81 FPKM/transcript) transcripts have remarkably much lower redundancy gauged by their gene expressions. The same has been observed in a number of snake venom gland transcriptomic studies, which reported >a thousand-fold higher expression of toxin genes in comparison to non-toxin and unidentified genes [27,28,29,30,31]. The high expression of toxin genes is in line with the venom gland’s function in synthesizing secretory proteins into the venom, while the non-toxin genes are mainly house-keeping genes for basic cellular functions. The high redundancy of toxin genes also reflects the existence of families of homologous toxins that complement each other in their biological functions or act synergistically in sum to produce the venom’s effect.

### 2.2. Overview of Toxin Gene Expression in C. rhodostoma Venom Gland Transcriptome

The 97 transcripts were subsequently classified into 16 protein families of snake venom toxins. After identifying redundant sequences, the number of unique toxin transcripts was further reduced to 92 (Table 2). Despite the very high overall expression of toxin genes in the transcriptome, the number of these genes is relatively small, and they are distributed with repetitions in a restricted set of protein families. In contrast to the generally perceived thought that snake venom contains myriad proteins, the transcriptomic profile and previous proteomic findings appear to be consistent with the dominant view of toxin recruitment through gene duplication and neofunctionalization, with consequent streamlining of venom functional complexity. Of these, 30 transcripts are identified as full-length protein-encoding transcripts (defined here as >90% coverage of the protein-coding region of the annotated protein sequences) (Table 3). Based on the FPKM as a quantitative parameter to gauge gene expression, the snake venom metalloproteinase (SVMP) was found to be the dominant toxin family in the *C. rhodostoma* venom gland transcriptome, accounting for 37.84% of all toxin transcriptions. This is followed by phospholipase A_2_ (PLA_2_, 29.02%), bradykinin-potentiating peptide/angiotensin-converting enzyme inhibitor-C-type natriuretic peptide (BPP/ACEI-CNP, 16.30%), and snake venom C-type lectin (snaclec, 10.01%). Toxins from these four families constitute 93.16% of the overall toxin FPKM (Figure 1). The rest of the toxin families identified from the venom gland transcriptome of *C. rhodostoma* are snake venom serine proteinase (SVSP, 2.81%), L-amino acid oxidase (LAAO, 2.25%), cysteine-rich secretory venom protein (CRiSP, 0.9%), 5′-nucleotidase (5′NT, 0.28%), phospholipase B (PLB, 0.25%), nucleobindin (NLB, 0.19%), nerve growth factor (NGF, 0.07%), vascular endothelial growth factor (VEGF, 0.05%), three-finger toxin (3FTX, 0.02%), aminopeptidase (APP, 0.01%), phosphodiesterase (PDE, 0.01%), and Kunitz-type serine proteinase inhibitor (KSPI, <0.01%) (Figure 1). 

The dominance of SVMP transcription (37.8% of all toxin FPKM) revealed in the *C. rhodostoma* venom gland transcriptome is in agreement with the high abundance of SVMP (41.2% of total venom proteins) found in the proteome of Malaysian *C. rhodostoma* venom [18]. Nonetheless, the expression levels of other toxin transcripts appear to be at variance with the protein abundances of toxins found in its venom proteome. For example, while the proteomic study found relatively high abundances of snaclec (26.3%), snake venom serine proteases (SVSP, 14.9%), and L-amino acid oxidase (LAAO, 7%) in the venom, the FPKM values, which indicate gene expression levels of the corresponding toxins, were only 10.1%, 2.6%, and 2.25%, respectively. Statistical analysis with the Pearson correlation test found no significant correlation between gene expression (current transcriptomic study) and protein abundance (proteomic study, [18]) (correlation coefficient *r* = 0.711, *p*-value > 0.05). The discrepancy observed is not unusual, as similar findings have been reported in a number of studies [28,29,32,33]. The causes of the no-correlation observed could be multifactorial and occur at different stages of protein synthesis. The process could be modulated by complex regulation of protein translation at different time points, varying synthesis rates, and half-lives that varied among the transcripts of different toxins [32,33]. The transcriptomic profile, therefore, is a snap-shot picture of toxin gene expression at day 4 post-venom milking, while the proteome represents the cumulation of various toxin proteins over an unspecified duration of venom production and storage in the glands. In addition, the differences could be due to varying sensitivities of detection methods used, different numbers of animals studied, and the fact that transcripts detected are not necessarily translated into proteins. Nevertheless, the dominance of SVMP reported in both data sets suggests these toxins are actively and consistently expressed for important biological functions of the venom (discussed below).

### 2.3. Toxin Gene Diversity and Implications for the Bioactivity of Snake Venom

The transcriptomic profile reveals substantial complexity in the toxin genes expressed by *C. rhodostoma*. The diversity of the toxin genes, though complex, appears to generally conform to a small subset of predictable gene families that are characteristic of Viperidae snakes. The following sections analyzed the sequence data of the toxins with correlation to their bioactivities. 

#### 2.3.1. Snake Venom Metalloproteinase (SVMP)

While snake venom toxins are constrained to a restricted number of protein families, each protein family typically contains multiple proteoforms (isoforms) that exhibit a breadth of structural diversity across taxonomically distinct and geographically distant snake species [32]. Snake venom metalloproteinase (SVMP) is one such example, which is arguably the most structurally and functionally diverse snake toxin superfamily. Based on the domain structure and molecular mass, SVMPs are conventionally classified into three main subgroups or classes: P-I, which is relatively small (~20–30 kDa) and consists of only the metalloproteinase domain; P-II (~30–60 kDa), characterized by the presence of a disintegrin domain in addition to the metalloproteinase domain; and P-III (>60 kDa), with the presence of a cysteine-rich domain in addition to the disintegrin-like and metalloproteinase domains [34,35]. In the present study, the SVMP family represents 34.87% of all toxin FPKM in the Malaysian *C. rhodostoma* venom gland transcriptome, where the subgroup P-I SVMP is the most abundant (26.51%), expressed at a higher level than the P-II SVMP (7.94%) and P-III SVMP (3.39%) (Figure 1). The dominance of P-I SVMP in the venom gland transcriptome is consistent with the proteomic finding of *C. rhodostoma* venom (of Malaysian origin), where the protein abundance of P-I SVMP was found to be 20.4% of total venom proteins. The P-II SVMP and P-III SVMP protein abundances, on the other hand, were reported to be 19.8% and 1%, respectively, in the venom proteome [18]. It may be speculated that the discrepancy between the exact values of transcripts and proteins is due to a different rate of mRNA synthesis at the time of sampling, resulting in a lower or higher transcript abundance in comparison to the abundance of mature proteins, which were accumulated over a longer period of time in the glands.

**Figure 2:**
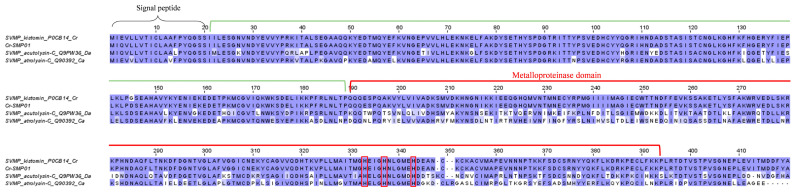
Multiple amino acid sequence alignment of Cr-SMP01 (P-I snake venom metalloproteinase, P-I SVMP kistomin) identified in *Calloselasma rhodostoma* venom gland transcriptome in comparison to selected P-I SVMP retrieved from UniProtKB database. black brace: signal peptide domain; green bracket: propeptide domain; red bracket: metalloproteinase domain. Highlighted in red boxes: amino acid residues involved in metal binding. Abbreviation: Cr—*Calloselasma rhodostoma*; Da—*Deinagkistrodon acutus*; Ca—*Crotalus atrox*. Color shading intensity of amino acid residues indicates conservation at the respective positions: the most conserved columns in each group have the most intense colors, and the least conserved are the palest. White background implies non-conserved amino acid residues.

Among the P-I SVMP transcripts, Cr-SMP01, which is a homolog to kistomin (UniProt ID: P0CB14), is the most highly expressed toxin in the *C. rhodostoma* venom gland (Table 3). Similarly, this protein was found to be the most abundant SVMP form in the *C. rhodostoma* venom proteome [18]. Huang et al. first isolated and characterized kistomin, an anti-platelet protease composed of 227 amino acid residues that selectively cleaves human platelet glycoprotein GPIb [36]. Kistomin was further shown to affect glycoprotein Ib-von Willebrand factor interaction, causing an anti-thrombotic effect [37], and hydrolyze glycoprotein VI (GPVI, the transmembrane receptor on platelets), thereby inhibiting the interaction between platelets and collagen [37]. Clinically, *C. rhodostoma* envenoming invariably results in hemorrhagic effects [13,14,15], consistent with the high expression of this hemorrhagin protein. Our multiple sequence analysis shows the Cr-SMP01 gene is nearly identical to kistomin, and it comprises the three domains of a signal peptide, a propeptide, and the metalloproteinase (Figure 2). Its signal peptide and propeptide domains are also highly similar to two well-characterized PI SVMPs from *Deinagkistrodon acutus* (acutolysin, UniProt ID: Q9PW36) and *Crotalus atrox* (atrolysin, UniProt ID: Q90392), demonstrating a high degree of conservation of these non-mature protein coding regions. The metalloproteinase domain, on the other hand, is variable, with multiple substitutions accumulated in these genes (Figure 2). These are coding genes for SVMP that are subject to the pressure of positive selection and the consequent accelerated evolution for adaptation to various ecological niches occupied by different species [38]. 

**Figure 3:**
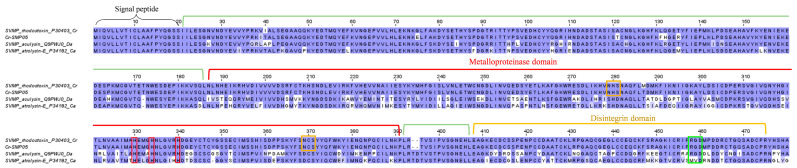
Multiple amino acid sequence alignment of Cr-SMP05 (P-II snake venom metalloproteinase, P-II SVMP rhodostoxin) identified in *Calloselasma rhodostoma* venom gland transcriptome in comparison to selected P-II SVMP retrieved from UniProtKB database. Black brace: signal peptide domain; green bracket: propeptide domain; red bracket: metalloproteinase domain; orange bracket: disintegrin domain. Highlighted in red boxes: metal binding site; highlighted in orange boxes: N-glycosylation sites; highlighted in green box: disintegrin motif. Abbreviation: Cr—*Calloselasma rhodostoma*; Da—*Deinagkistrodon acutus*; Ca—*Crotalus atrox*. Color shading intensity of amino acid residues indicates conservation at the respective positions: the most conserved columns in each group have the most intense colors, and the least conserved are the palest. White background implies non-conserved amino acid residues.

The major P-II SVMP gene, annotated as Cr-SMP05 in the *C. rhodostoma* venom gland, has a high sequence identity matching to the protein “zinc metalloproteinase/disintegrin” (UniProt ID: P30403) (Table 3), which is more commonly referred to as rhodostoxin or rhodostomin in vast publications [39,40,41,42]. The respective toxins (rhodostoxin and rhodostomin) are, nonetheless, distinct, and each represents an individual cleavage product of the common P-II SVMP precursor (Figure 3). Au et al. [39] cloned the platelet aggregation inhibitor rhodostomin and showed that its 68-amino acid sequence is located at the carboxyl terminus of a much larger precursor polypeptide, which was later verified as part of a true hemorrhagin protein containing a metalloproteinase domain as in the SVMP of the PI class [40,43]. As with rhodostomin’s precursor, the transcript Cr-SMP05 possesses the characteristic peroxisomal targeting sequence (Ser-His-Ala) at the C-terminus (Figure 3). Moreover, the signature motif of disintegrin, i.e., the RGD (Arg-Gly-Asp) motif, is found (highlighted in green) in Cr-SMP05, rhodostomin (UniProt ID: P30403), and aculysin (UniProt ID: Q9WJ0, derived from another basal crotaline of the Old World, *D. acutus*) but, intriguingly, not in atrolysin-E (UniProt ID: P34182, derived from the New World crotaline, *Crotalus atrox*). The RGD motif is deemed crucial for specific binding between disintegrin and integrin receptors [44], although a number of disintegrins have been found to have variable sequences that do not necessarily conform to the RGD motif [41]. On the other hand, the metalloproteinase-containing domain of Cr-SMP05 resembles the sequence of rhodostoxin, the hemorrhagin derived from the same precursor gene as for rhodostomin (Figure 3). Rhodostoxin is the first four-disulfide proteinase reported among all known venom metalloproteinases [40]. Two N-glycosylation sites have been identified (highlighted in orange) in the metalloproteinase domain of Cr-SMP05, consistent with those reported in rhodostoxin [40] (Figure 3). 

Rhodostoxin is a major hemorrhagin in *C. rhodostoma* venom that exhibits strong proteolytic and potent hemorrhagic activity (minimum hemorrhage dose, MHD = 0.13 µg intradermally in mice), although its lethality is low (non-lethal even at an intravenous dose >6 µg/g in rodents) [45]. Its target site of action and mechanism of action have not been investigated in depth, although based on its metalloproteinase domain, one can deduce that it acts in a manner similar to other hemorrhagins by damaging the collagenous basement membrane and destabilizing the vascular integrity, leading to blood extravasation and hemorrhagic effect [46,47]. Rhodostomin, on the other hand, has received more research attention over the years, presumably due to its pharmaceutical potential as an anti-platelet and anti-proliferative agent. Rhodostomin blocks the binding of fibrinogen to the fibrinogen receptor, the glycoprotein IIb/IIIa complex of an activated platelet [39]. It also blocks basic fibroblast growth factor (bFGF) and the integrin ɑ_v_ß_3_ receptor, the blockade of which extends its anti-angiogenesis property for tumor growth suppression [42]. 

Consistent with earlier cloning studies, the current transcriptomic finding in *C. rhodostoma* venom gland supports the view that hemorrhagin metalloproteinases (e.g., rhodostoxin) and anti-platelet disintegrins (e.g., rhodostomin) share common precursors [39,48]. Disintegrins, including rhodostomin, are typically isolated as individual low molecular mass proteins (MW 6–7 kDa) from crude venoms [18,49,50], while stand-alone transcripts have not been identified in venom gland transcriptomes, implying they are generated by proteolysis of translated P-II SVMPs [51]. The yields of metalloproteinase and disintegrin cleaved thereof, obviously, do not exist at a balanced ratio of 1:1 in any proteomic study of snake venom [18,49,50]. The natural occurrence and abundance of disintegrins in any viperid venom are usually low relative to SVMP and are virtually non-existent in elapid venoms [52]. The mechanism that determines the rate and extent of disintegrin release from the cleavage of P-II SVMP in a venom gland remains unresolved. Nonetheless, the derivation of the hemorrhagin and anti-platelet toxin from the same precursor gene and their co-existence in an intact large protein suggest an important synergistic function in hemotoxic envenoming caused by the viperids. From a pathological point of view, the action of metalloproteinase (destruction of the basal membrane and vascular stability) coupled with that of disintegrin (anti-platelet plug formation) at the venom injection site would contribute to enhanced bleeding. In real envenoming, the local hemorrhagic activity would be exacerbated by systemic coagulopathy (induced by procoagulant and anticoagulant toxins) [14], thrombocytopenia, and platelet dysfunction (caused by snaclecs) [53,54]. Hypothetically, the stand-alone disintegrins could be absorbed from the bite wound and thus contribute to the systemic effect on platelets.

**Figure 4:**
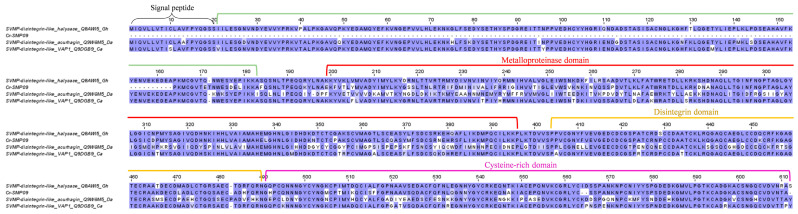
Multiple amino acid sequence alignment of Cr-SMP09 (P-III snake venom metalloproteinase, P-III SVMP) identified in *Calloselasma rhodostoma* venom gland transcriptome in comparison to selected P-III SVMP retrieved from UniProtKB database. Black brace: signal peptide domain; green bracket: propeptide domain; red bracket: metalloproteinase domain; green bracket: disintegrin domain; purple bracket: cysteine-rich domain. Abbreviation: Gh—*Gloydius halys*; Da—*Deinagkistrodon acutus*; Ca—*Crotalus atrox*. Color shading intensity of amino acid residues indicates conservation at the respective positions: the most conserved columns in each group have the most intense colors, and the least conserved are the palest. White background implies non-conserved amino acid residues.

The SVMP of P-III class have a higher molecular mass (typically > 60 kD) and are most intriguing in terms of structural complexity and function variety [55]. In addition to the typical “SVMP + disintegrin-like + cysteine-rich” composition, some P-III SVMPs are found with an additional lectin-like domain that gives rise to unique substrate specificity and, hence, functionality. The present study uncovered a novel SVMP transcript, Cr-SMP09, from the venom gland transcriptome of *C. rhodostoma*, which is most likely the first identified P-III SVMP gene in this species. The sequence of Cr-SMP05 shows the highest identity matched to halysase (UniProt ID: Q8AW15), an SVMP-disintegrin-like protein from *Gloydius halys* (Figure 4). The identification of this transcript as the main P-III SVMP of *C. rhodostoma* verifies previous proteomic findings in which peptide fragments were matched to the SVMP-disintegrin-like halysase instead of one belonging to other species [17,18]. The transcript identified, unfortunately, lacks complete information for the signal peptide and propeptide, but it retains the full sequence of metalloproteinase, disintegrin-like, and cysteine-rich domains (Figure 4). Comparing homologous P-III SVMP sequences among Cr-SMP09, halysase, acurhagin (UniProt ID: Q9W6M5, from *D. acutus*), and VAP1 (UniProt ID: Q9DGB9, from *C. atrox*), structural variation is high in acurhagin, where multiple substitutions were found throughout the functional coding domain, suggesting higher evolutionary events and possibly a more adaptive function for *D. acutus* (Figure 4). In *C. rhodostoma*, the expression of Cr-SMP09 is low (3.32% of all toxin FPKM), consistent with the low protein abundance of P-III SVMP in our proteomic study (~1% of total venom proteins). The low protein abundance has been a limitation to the purification and characterization studies of this novel protein. 

#### 2.3.2. Phospholipase A_2_ (PLA_2_)

Snake venom PLA_2_s (svPLA_2_s) exhibit a wide array of pharmacological activities, including myotoxicity, nephrotoxicity, anti-coagulant effects, and inflammation, owing to their structural versatility [56,57]. Used to be thought of as a ubiquitous group of enzymatic toxins in all snake venoms, svPLA_2_ abundances are now known to vary remarkably from a high of >60% in Russell’s Viper venoms [58,59] to virtually none in the venoms of *Naja nivea*, *Naja annulifera,* and *Naja senegalensis* (subgenus: *Uraeus*) [60,61,62]. Previous studies showed *C. rhodostoma* venom has low PLA_2_ enzymatic activity [45,63], consistent with its proteome, in which PLA_2_ constitutes only 4.4% of the total venom proteins [18]. The apparently higher abundance of PLA_2_ transcripts in the venom gland transcriptome is puzzling, suggesting complex regulation of the mRNA half-life and protein translation that cannot be simplified by a single snapshot of the transcriptomic profile. In fact, the disproportionate expression between PLA_2_ transcripts and proteins is not uncommon, as reported in a number of venom gland transcriptomics studies for *Naja kaouthia* [27,64], *Naja sumatrana* [29,65], *Ophiophagus hannah* [28], *Hydrophis curtus* [31,66], and *Calliophis bivirgata* [30,67], when venom glands were harvested 3–4 days post-venom milking. 

**Figure 5:**
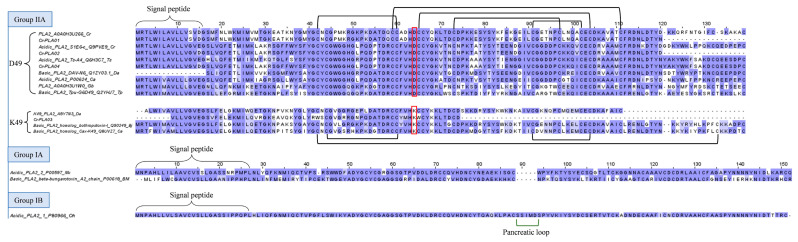
Multiple amino acid sequence alignment of secretory phospholipases A_2_ (PLA_2_s) from *C. rhodostoma* venom gland transcriptome in comparison to PLA_2_s of related species, categorized according to group (II, IA, and IB) and subtype (D49, aspartic acid at 49th amino acid residue, and K49, lysine at 49th amino acid residue). Black brackets: disulfide bonds; highlighted in red box: D49 or K49 residue. Abbreviation: Cr—*Calloselasma rhodostoma*; Ts—*Trimeresurus stejnegeri*; Da—*Deinagkistrodon acutus*; Ca—*Crotalus atrox*; Gb: *Gloydius brevicaudus*; Tp: *Trimeresurus puniceus*; Bj—*Bothrops jararacussu*; Nk—*Naja kaouthia*; Bm—*Bungarus multicinctus*; Oh: *Ophiophagus hannah*. Color shading intensity of amino acid residues indicates conservation at the respective positions: the most conserved columns in each group have the most intense colors, and the least conserved are the palest. White background implies non-conserved amino acid residues.

The present study identifies a number of acidic svPLA_2_ transcripts in the *C. rhodostoma* venom gland transcriptome, notably Cr-PLA01, Cr-PLA02, Cr-PLA03, and Cr-PLA04, which form the main bulk of PLA_2_ transcripts (Table 2). The mRNAs uncover the PLA_2_ genes with high expression (FPKM >10,000), and structurally, they belong to Group IIA PLA_2_ as most viperid PLA_2_s do (Figure 5). Cr-PLA01 and Cr-PLA02 are matched with the highest homology to previously deposited *C. rhodostoma* PLA_2_ sequences of A0A0H3U266 (uncertain origin) and Q9PVE9 (Thailand, [68]), respectively. Cr-PLA03 and Cr-PLA04 are homologous to PLA_2_s from *D. acutus* (ABY7N3, uncertain origin) and *Trimeresurus stejnegeri* (Q6H3C7, Taiwan, [69]), respectively. Variations are noted among the matched PLA_2_ pairs, in particular for Cr-PLA01 and Cr-PLA03. Cr-PLA01 (and the homologous A0A0H3U266) appears to have a higher mutation rate, exhibiting a considerably variable N-terminal sequence with several non-conserved substitutions: ^1^XXXNLWXXXXVMXXXEATKNXXMXXXNXXPMK^32^ (Figure 5). Cr-PLA03 has relatively lower variability except for an important substitution at the 49th amino acid residue, in which aspartic acid (D) is substituted for lysine (K), classifying it as a K49 PLA_2_. The presence of aspartic acid at this position (D49) imparts the catalytic activity critical for PLA_2_ enzymatic reaction [70], and it is highly conserved across lineages, including *D. acutus* (Q1ZY03), *T. stejnegeri* (Q6H3C7), *T. puniceus* (Q2YHJ7), and *Gloydius brevicaudus* (A0A0H3U1W0) of the Old World, as well as *Crotalus atrox* (P00624) and *Bothrops jararacussu* (Q90249) of the New World (Figure 5). The substitution of this amino acid (D) for lysine (K) in Cr-PLA03 is anticipated to result in a complete loss of phospholipase activity while gaining myotoxic activity, a toxic trait of neofunctionalization [71]. Clinically, systemic myotoxicity is rarely reported in *C. rhodostoma* envenoming, although the various PLA_2_ enzymes expressed may act in synergism with other toxins, contributing to inflammation, cytolytic, and tissue-destructive effects. Cr-PLA03, a novel K49 PLA_2_ gene in *C. rhodostoma*, is highly homologous to the K49 variant found in *D. acutus* (A8Y7N3, Taiwan) and, to a lesser degree, K49 PLA_2_s from the New World pit vipers (e.g., Q90249, Q8UV27) (Figure 5), which are more clinically toxic and extensively studied. The finding supports the recruitment of K49 PLA_2_ in the Asiatic basal crotalines and implies these are orthologous genes, possibly with multiple ancestries. 

Notwithstanding the sequence variability, these viperid PLA_2_ sequences (Group II) show highly conserved 7 disulfide bonds and an extended C-terminal tail, with the absence of the elapid or pancreatic loop found in elapid svPLA_2_s, which are structurally classified under Groups IA and IB (Figure 5). 

#### 2.3.3. Bradykinin-Potentiating Peptide (BPP)/Angiotensinogen-Converting Enzyme Inhibitor (ACEI), and Natriuretic Peptide (NP)

**Figure 6:**
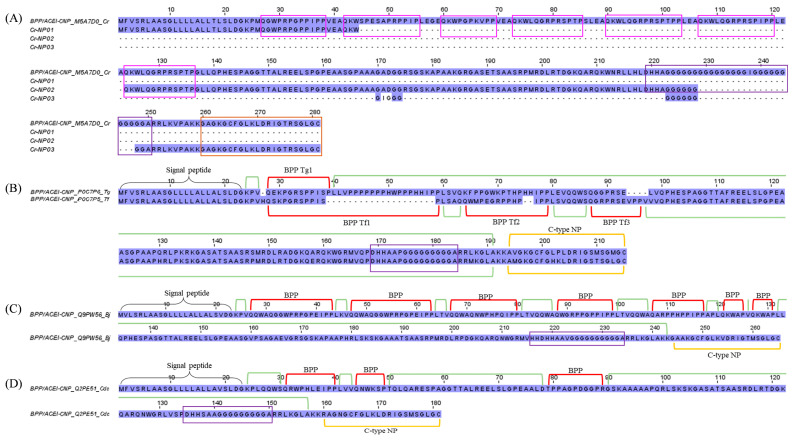
Multiple amino acid sequence alignment of bradykinin-potentiating peptides/angiotensin-converting enzyme inhibitors—C-type natriuretic peptide (BPP/ACEI-CNP) from *C. rhodostoma* venom gland transcriptome in comparison to BPP/ACEI-CNPs of related species. (**A**) CNP transcripts were aligned with BPP/ACEI-CNP of *C. rhodostoma* (UniProt ID: M5A7D0). (**B**) Alignments of BPP/ACEI-CNP from *Trimeresurus gramineus* (UniProt ID: P0C7P6) and *Trimeresurus flavoviridis* (UniProt ID: P0C7P5). (**C**) Alignment of BPP/ACEI-CNP from *Bothrops jararacussu* (UniProt ID: Q9PW56). (**D**) Alignment of BPP/ACEI-CNP from *Crotalus durissus collilineatus* (UniProt KB: Q2PE51). Highlighted in pink boxes: probable bradykinin-potentiating peptide (BPP) domains of *C. rhodostoma*; highlighted in purple boxs: spacer domains; highlighted in brown: probable C-type natriuretic peptide (CNP) of *C. rhodostoma*. Black brace: signal peptide domain; green bracket: propeptide domain; red bracket: BPP domain; yellow bracket: C-type NP. Abbreviation: BPP Tg1—Bradykinin-potentiating peptide *Trimeresurus gramineus* 1; BPP Tf1/2/3—Bradykinin-potentiating peptide *Trimeresurus flavoviridis* 1/2/3; Cr—*Calloselasma rhodostoma*; Tg—*Trimeresurus gramineus*; Tf—*Trimeresurus flavoviridis*; Bj—*Bothrops jararacussu*; Cdc—*Crotalus durissus collilineatus*. Color shading intensity of amino acid residues indicates conservation at the respective positions: the most conserved columns in each group have the most intense colors, and the least conserved are the palest. White background implies non-conserved amino acid residues.

Bradykinins (belong to the kallikrein-kinin system) and natriuretic peptides (NPs) are endogenous mammalian peptides involved in the homeostasis of body fluid and regulation of blood pressure [72]. Snake venoms would have evolved to target, mimic, and disrupt these systems in the mammalian prey, subjecting it to a secondary effect of the venom, e.g., a significant drop in blood pressure that ultimately leads to the subduing of the prey [73,74,75]. Bradykinin-potentiating peptides (BPPs) and C-type NPs (CNPs) are two well-characterized vasoactive blood pressure-modulating snake toxins found especially among pit vipers [74]. The occurrence of these peptides is mostly based on findings from cDNA sequencing, transcriptomics, or genomics (e.g., *Bothrops jararaca*, *Trimeresurus flavoviridis*, *Trimeresurus gramineus,* and *Agkistrodon halys blomhoffi* [76]; *Bothrops jararaca* [77]), while detection at the protein level has been scarce, presumably due to the peptides’ low abundances and small molecular sizes that curtail detection (e.g., *Trimeresurus flavoviridis* [78]; *Bitis gabonica rhinoceros* [79]). The present study identified a substantial level of BPP/ACEI-CNP transcripts in the *C. rhodostoma* transcriptome (16.3% of all toxin FPKM), with homolog sequences matched to a multi-domain gene (UniProt ID: M5A7D0) coding for a putative BPP/ACEI-CNP precursor, which was previously deposited in the databank in 2021 (Figure 6). Unfortunately, as of today, no such protein has been isolated from *C. rhodostoma* venom, and no characterization has been done to locate the domains of BPP, ACEI, or CNP in the gene. 

To gain insight into its gene structure, we compare the transcript sequences with homologous BPP/ACEI-CNP genes retrieved from the databank. Using the sequence from *Trimeresurus gramineus* (P0C7P6) as a classic template, this “composite” precursor gene has a signal sequence (a.a. residues 1–25) at the N-terminus, a pro-peptide, and a modified residue (residues 24–26), followed by the BPP peptide coding region (residues 27–38), which is then spaced by another long propeptide or linker sequence (residues 39–186) with an unidentified function. This is followed by another processing signal prior to the CNP sequence (residues 189–210) toward the C-terminus (Figure 6). It should be noted that in many other species, e.g., *Protobothrops flavoviridis* (P0C7P5), *Bothrops jararaca* (Q9PW56), and *Crotalus durissus collilineatus* (Q2PE51), more than one BPP coding region has been identified in their respective orthologous genes, embedded variably within the long spacer prior to the signal sequence of CNP (Figure 6). Our transcriptome findings revealed at least one BPP/ACEI and one CNP sequence that are unique to *C. rhodostoma* of Malayan origin. By comparison to the other sequences, we managed to annotate the putative BPP- and CNP-coding regions in the transcripts and the precursor gene of *C. rhodostoma* (M5A7D0). Cr-NP01 has a propeptide identical to M5A7D0, and both share an identical BPP of 12 amino acid residues with an N-terminal sequence of QGWPRPGPPIPP. Classically, the BPPs have pyroglutamic acid (E, a post-translational modification normally annotated as glutamine in the precursor gene sequence) at the N-terminus, and a notable high content of proline (P) residues [80], which gives them some resistance to hydrolysis by aminopeptidases, carboxypeptidases, and endopeptidases [81]. We further predicted a few additional BPP-coding domains in the precursor gene M5A7D0 based on the canonical BPP modular motif and identified another BPP with the sequence QKWKQGRPRSPTP (13 amino acid residues) from the transcript Cr-NP02 (Figure 6). This was followed by a long spacer gene that carries an intriguingly high content of repeated glycine (G) residues within a well-conserved poly-His-poly-Gly region. The *C. rhodostoma* CNP sequence (GAGKGCFGLKLDRIGTRSGLGC) was discovered in the transcript Cr-NP03. Preceded by the conserved short signal peptide AKK, the CNP shares high homology with those of other pit vipers, consisting of 28 amino acid residues arranged in three highly conserved segments: GCFG, DRIG, and SGLGC (C-terminus) (Figure 6). The first five residues of their N-termini undergo substitution, but the overall structural constraint already established leaves little room for “improvisation”. The natural occurrence of CNP is a redundancy to the BPPs, as both peptides ultimately promote the same biological effect, i.e., vasorelaxation induced by increased guanylate cyclase levels in the vascular smooth muscle cells [78]. While these vasodilating peptides can contribute to systemic hypotension, locally they may promote capillary permeability, thereby expediting the diffusion and spread of other toxins present in the venom. The exact pathophysiological role of BPP/ACEI and CNP in *C. rhodostoma* venom is as elusive as its gene origin and warrants further exploration.

#### 2.3.4. Snake C-Type Lectins

**Figure 7:**
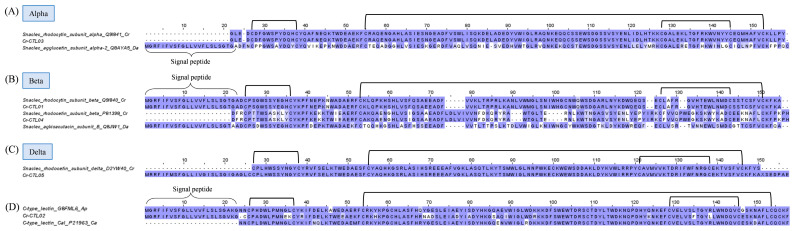
Multiple amino acid sequence alignment of Cr-CTL01–04 (snake C-type lectins) identified in *Calloselasma rhodostoma* venom gland transcriptome in comparison to snake CTLs selected from UniProtKB database. Snaclecs are categorized according to their alpha subunit (**A**), beta subunit (**B**), and delta subunit (**C**), while sugar-binding lectins are shown in (**D**). Black brackets depict disulfide bonds. Abbreviation: Cr—*Calloselasma rhodostoma*; Da—*Deinagkistrodon acutus*; Ap—*Agkistrodon piscivorus*; Ca—*Crotalus atrox*. Color shading intensity of amino acid residues indicates conservation at the respective positions: the most conserved columns in each group have the most intense colors, and the least conserved are the palest. White background implies non-conserved amino acid residues.

Snake C-type lectins are categorized into snake lectins and snake C-type lectin-like proteins (snaclecs). While snake lectins are classic sugar-binding proteins, snaclecs are usually unable to recognize carbohydrates as they lack the Ca^2+^ ion binding loop involved in sugar binding [82,83]. Snake lectins are 26–28 kDa homodimers, whereas snaclecs are more structurally complex, consisting of loop-swapping heterodimers formed by homologous α- and β-subunits, each with a molecular mass ranging from 13 to 18 kDa [84]. Snaclecs are functionally more relevant to toxicity than lectins, in particular for viperid snakes such as *C. rhodostoma.* Snaclecs, in the major forms of rhodocytin and rhodocetin, have been found to constitute up to 26% of its total venom proteome [18]. In the current study, the overall expression of snaclecs in the *C. rhodostoma* venom gland is 10.01% of the total FPKM of all toxins (Figure 1). Two complete sequences of snaclecs (Cr-CTL03 and Cr-CTL01) matching identically to the rhodocytin subunit ɑ (UniProt ID: Q9I840) and rhodocytin subunit ß (UniProt ID: Q9I841), respectively, were identified from the venom gland transcriptome (Figure 7). Rhodocytin, also known as aggretin, is a 29 kDa heterodimer snaclec that interacts with the CLEC-2 (C-type lectin 2) receptor on platelets, inducing platelet aggregation [85,86]. The subunits Cr-CTL03 and Cr-CTL01 of *C. rhodostoma* show a lesser degree of sequence identity to the corresponding subunits of snaclec from *D. acutus*. Multiple substitutions contributing to the heterogeneity were observed between the two species. Six cysteine residues and three disulfide links are nonetheless well-conserved in each of the monomeric subunits (Figure 7). 

The other two transcripts of lower expression, Cr-CTL04 and Cr-CTL05, mark the expression of rhodocetin, a snaclec that binds to GPIb and inhibits VWF-dependent platelet aggregation [83,87]. Rhodocetin is, in fact, an α_2_β_1_ integrin-specific antagonist and a hetero-tetrameric molecule composed of alpha-beta and gamma-delta subunits arranged orthogonally in a cruciform pattern [88]. The sequence of Cr-CTL04 differs from that of the rhodocytin beta subunit Cr-CTL01 by acquiring an additional segment of LDLVI while losing some residues in comparison to rhodocytin (Figure 7). Cr-CTL05 is identical to the delta subunit of rhodocetin (Figure 7), while rhodocytin as a dimer has no comparable delta subunit. All subunits of rhodocetin and rhodocytin, nonetheless, shared conserved cysteine residues and disulfide bonds, which are responsible for the overall well-conserved and robust structure of snaclecs. Apparently, both snaclecs appear to share the same target receptor (platelet), but rhodocetin interacts with α_2_β_1_ integrin (the major collagen receptor on the platelet) in an RGD-independent manner, hampering platelet activation [89]. Simply put, while rhodocytin is a platelet aggregator, rhodocetin inhibits platelet aggregation. This is again a redundancy of venom function—although the actions on platelets seem to be opposing, the resultant effect is thrombocytopenia with dysfunctional platelets as part of the pathophysiology of *C. rhodostoma* envenoming. 

In addition, the *C. rhodostoma* venom gland transcriptomics reveals a novel transcript of snake C-type lectin (Cr-CTL02), which is homologous to venom lectins from *Agkistrodon piscivorus leucostoma* and *Crotalus atrox* (Figure 7). The role of galactose-binding snake lectins in *C. rhodostoma* envenoming is unclear, although experimentally, lectins from many New World species were found to exhibit hemagglutinating, inflammatory, and platelet-aggregating activities [90].

#### 2.3.5. Snake Venom Serine Proteinase (SVSP)

**Figure 8:**
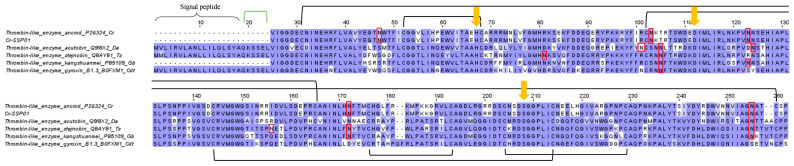
Sequence alignment of Cr-SSP01 (thrombin-like snake venom serine proteinase, TL-SVSP) identified in *Calloselasma rhodostoma* venom gland transcriptome in comparison to selected SVSPs from UniProtKB database. Black bracket: disulfide bond; green bracket: propeptide. Highlighted in red boxes: putative N-linked glycosylation site. Yellow arrows indicate the conserved catalytic triad of histidine (His57), aspartate (Asp102) and serine (Ser195) with numbering based on the chymotrypsinogen system. Abbreviation: Cr—*Calloselasma rhodostoma*, Da—*Deinagkistrodon acutus*; Ts—*Trimeresurus stejnegeri*; Gb—*Gloydius brevicaudus*; Cdt—*Crotalus durissus terrificus*. Color shading intensity of amino acid residues indicates conservation at the respective positions: the most conserved columns in each group have the most intense colors, and the least conserved are the palest. White background implies non-conserved amino acid residues.

Snake venom serine proteinases (SVSPs) catalyze a broad range of reactions targeting the coagulation cascade, kallikrein-kinin and fibrinolytic systems, complement system, endothelial cells, and platelets, all of which eventually augment the hemotoxic effect of viperid envenoming [91]. The SVSP abundance in *C. rhodostoma* venom proteome is well established to be around 15–25% of total venom proteins [17,18,20], while in the current transcriptomic study, the SVSP transcript level is disproportionately low (2.8% of all toxin FPKM) (Figure 1). The dominant transcript identified (Cr-SSP01) corresponds to the major SVSP expressed in the venom proteome, i.e., ancrod (or arvin) [18], which belongs to the thrombin-like SVSP (TL-SVSP) subgroup. The sequence of Cr-SSP01 matches identically to the ancrod of *C. rhodostoma* (uncertain locality) reported in 1992 [92] but has a low degree of sequence identity when compared with TL-SVSP from *D. acutus* (acutobin, 60.1%), *Gloydius brevicaudus* (kangshuanmei, 64.0%), *Trimeresurus (Viperidovipera) stejnegeri* (stejnobin, 63.4%), and *Crotalus durissus terrificus* (gyroxin, 61.3%) (Figure 8). Despite the variations, these TL-SVSP of various pit viper genera share the conserved catalytic triad of serine (Ser195), histidine (His57), and aspartate (Asp102) (residue numbering according to the chymotrypsinogen system) (Figure 8) [93]. As reported previously, ancrod was heavily glycosylated [94], consistent with our observation of five N-linked glycosylation sites revealed in Cr-SSP01 (Figure 8). Stejnobin too has five N-linked glycosylation sites, while acutobin and kangshuanmei have four, and gyroxin has only one. Glycosylation may play an important role in the maintenance of homeostasis within the gland lumen by improving protein solubility, protection from proteolytic attack, quality control of protein folding, prolonging proteins’ plasmatic half-life, target site recognition, and modulating the immunogenicity of protein [95,96,97,98].

TL-SVSPs are functionally similar to thrombin in some ways but are also dissimilar in many aspects (hence, “thrombin-like enzyme” is a slight misnomer). Ancrod represents a classical TL-SVSP that has been extensively studied and even clinically trialed as an anti-coagulant, though with a somewhat discouraging outcome [99,100]. Consumptive coagulopathy with defibrination is the major hemotoxic and potentially fatal effect of *C. rhodostoma* envenoming. This is produced by the cleavage of the fibrinogen α chain, releasing fibrinopeptides A, AP, and AY while leaving fibrins to form tenuous micro-thrombi that are readily dissolved [53]. Unlike thrombin, most TL-SVSPs do not activate factor XIII to stabilize the thrombus. The repeated dissolution of the tenuous clots in the background of continuous degradation of fibrinogen eventually leads to venom-induced consumptive coagulopathy, and the consequent massive bleeding, hypovolemic shock, hypo-perfusion, and multi-organ failure [101]. 

#### 2.3.6. L-Amino Acid Oxidase (LAAO)

**Figure 9:**
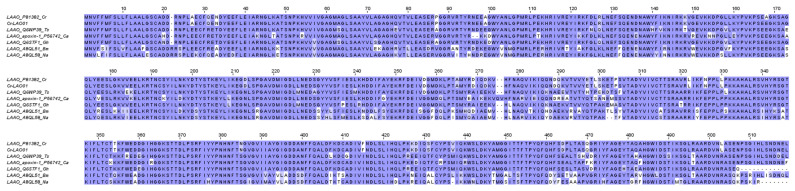
Sequence alignment of Cr-LAO01 (L-Amino acid oxidase, LAAO) identified in *Calloselasma rhodostoma* venom gland transcriptome in comparison to selected LAAO from UniProt KB database. Abbreviation: Cr—*Calloselasma rhodostoma*; Ts—*Trimeresurus stejnegeri*; Ca—*Crotalus atrox*; Gh—*Gloydius halys*; Bm—*Bungarus multicinctus*; Na—*Naja atra*. Color shading intensity of amino acid residues indicates conservation at the respective positions: the most conserved columns in each group have the most intense colors, and the least conserved are the palest. White background implies non-conserved amino acid residues.

L-Amino acid Oxidases (LAAO) are flavoenzymes that catalyze the stereo-specific oxidative deamination of L-amino acids to produce α-ketoacids, ammonia, and hydrogen peroxide (H_2_O_2_), the last of which is believed to account for the diverse toxicity of the enzyme, including hemorrhagic, hemolytic, anti-microbial, cytotoxic, and inflammatory activities [102,103]. The present study reveals a low level of LAAO transcripts in the *C. rhodostoma* venom gland transcriptome (2.25% of all toxin total FPKM), represented by one mRNA with full sequence (Cr-LAO01). The sequence of Cr-LAO01 is identical to the reported *C. rhodostoma* L-amino acid oxidase (UniProt ID: P81382) as shown in Figure 9, covering the three flavine adenine dinucleotide (FAD) binding sites and three substrate binding sites [104] (Figure 9). The abundance of LAAO in *C. rhodostoma* venom proteome is approximately 7% [18]. The pathological role of this toxin in *C. rhodostoma* envenoming is unclear but could be related to local inflammatory responses as it stimulates neutrophil activation and the production of inflammatory mediators [105].

Comparison of Cr-LAO01 with LAAOs from different snake genera reveals well-conserved sequences among the viperids, while the elapid LAAOs appear to be relatively more variable (sequence identity well below 80%) (Figure 9). In general, LAAO abundances are much lower and sometimes undetected in the venoms of elapid snakes, with the exception of King Cobra (*Ophiophagus hannah*), whose venom contains LAAO at >5% of total venom proteins [106].

### 2.4. Low-Abundance Toxin Transcripts 

#### 2.4.1. Toxins Detected in Both Venom Gland Transcriptome and Venom Proteome

In the *C. rhodostoma* venom proteome, a small amount of cysteine-rich secretory protein (CRiSP) was detected (1% of total venom proteins) [18], comparable to the transcript level of CRiSP (0.90% of all toxin total FPKM) found in this study (Figure 1). A full sequence of novel CRiSP (Cr-CRP01) was uncovered for this species. Most likely due to limited information on CRiSP for closely related viperids in the database, Cr-CRP01 was matched with the highest homology to the CRiSP LCCL domain (UniProt ID: VBN17) from the elapid *Ophiophagus hannah* (Supplementary File S2). Being cysteine-rich, as the name implies, these toxins have a highly conserved pattern of 16 cysteine residues. The toxicity is unclear, while they can cause blockage of calcium channels to inhibit smooth muscle contraction [107]. 

The study further unveils the gene expression of phosphodiesterases (PDE, a nuclease family) and 5′-nucleotidases (5′NT, a nucleotidase family) in the *C. rhodostoma* venom gland. The transcript levels of PDE and 5′NT were found to be low at 0.01% and 0.28% of all toxin total FPKM, respectively. In the venom proteome of *C. rhodostoma*, these were known to be low-abundance proteins (> 1% of total venom proteins) [18], and their function is putatively associated with the dissemination of venom toxins in the prey (or snakebite victims) [108]. PDE, as an exonuclease, cleaves DNA in a 3′,5′-direction, releasing 5′-mononucleotides that serve as nucleotide substrates for 5′NTs, which in turn liberate free nucleosides. The generation of purinic nucleosides (mainly adenosine) and other purine derivatives could lead to vasodilation and low blood pressure (facilitating prey immobilization), inflammation with enhanced toxin dissemination, and possible blockade of some neurotransmitters, as in the sympatholytic activity of adenosine [108]. Furthermore, the degradation of ADP and ATP by PDE may theoretically impair platelet aggregation activity at the wound site. Despite the low expressions of these enzyme toxins, the present study successfully uncovered the full-length sequences of PDE and 5′NT of *C. rhodostoma* (Supplementary File S2), which have never been characterized in depth. The sequences, representative of a basal pit viper from Asia, are matched to those of the New World pit viper (*Crotalus* spp.), whose PDEs and 5′NTs were more extensively studied and whose full sequences are readily available. Notwithstanding the distant phylogeographical relationship between *C. rhodostoma* and these New World pit vipers, a high degree of homology is found across these venom enzymes. There is very little or practically no important substitution observed in these orthologous genes of PDEs and 5′NTs in spite of their high molecular masses (>100 kDa). The finding suggests these proteins, though poorly expressed, are highly conserved with low evolution rates across different lineages. Presumably, these genes maintain the basic or secondary function of venom but are not subject to intense selective pressures compared with key toxins such as SVMP and SVSP, which are vital for predation. Minimum mutations involving these venom genes may help maintain existing beneficial phenotypes while promoting the rapid evolution of advantageous traits without interference from deleterious mutations. 

In proteomic studies, phospholipase B (PLB), nerve growth factor (NGF), and aminopeptidase (APP) have been detected at very low abundances (< 1% of total venom proteins, respectively) in *C. rhodostoma* venom [18,21]. The present study unveils the toxin genes of these minor proteins, and showed that the transcriptions of these genes are indeed low: PLB, 0.25%; NGF, 0.07%; APP, 0.01%, of all toxin total FPKM (Table 2). PLB cleaves ester linkages of membrane glycerophospholipids at both positions *sn-1* and *sn-2* [109], and this may be the basic mechanism by which venom PLB exerts its strong hemolytic and cytotoxic activities [110,111,112]. The full sequence of *C. rhodostoma* venom PLB is matched with high homology (>90% sequence identity) to that of *Bothrops moojeni*, indicating well-conserved conversation of this protein in snake venoms despite distant phylogenetic relationship (Supplementary File S2).

The full sequence of a snake venom NGF gene was discovered in the *C. rhodostoma* venom gland transcriptome. The sequence is nearly identical to that of *Protobothrops flavoviridis* (UniProt ID: B1Q3K2) (Supplementary File S2). The pathophysiological role of NGF, a key member of the neurotrophin family in snakebite envenoming is vague, but this non-enzymatic toxin may have an ancillary function in preserving the toxic cocktail of venom by inhibiting venom metalloproteinase-dependent proteolysis [113]. Sunagar et al. [114] demonstrated that venom-secreted NGFs have characteristics consistent with the typical accelerated molecular evolution of venom components driven by positive selection, suggesting their participation in prey envenomation. 

One APP gene was also found in this study for *C. rhodostoma*, identified based on its full sequence, which is homologous to that of *Ovophis okinavensis* (Supplementary File S2). APP belongs to metalloproteases, but little is known regarding its function and how it differs from the more widely studied SVMPs. Earlier, the characterization of an APP isolated from the venom of *Bitis arientans* (an African viper) suggested this enzyme could alter blood pressure and impair brain function in snakebite victims [115]. 

It should be noted that some of the putative toxins described herein as low-abundance venom constituents could be of cellular origin. While the isolated proteins demonstrated various pharmacological activities as described, the toxic effect may not be well defined in envenomation. Further studies are needed to characterize their pathophysiological roles so that new treatments can be innovated to abate the related toxicity.

#### 2.4.2. Toxins Detected Exclusively in Venom Gland Transcriptome

Previous studies have shown that not all toxin transcripts detected in snake venom gland transcriptomes are correspondingly translated and detected in the venom proteomes [28,32,33]. In the present study, we detected low-abundance transcripts of genes whose products have never been reported at the protein level in any biochemical and proteomic study of *C. rhodostoma* venom [17,18,19,21]. These are genes putatively coding for nucleobindin (NCB), vascular endothelial growth factor (VEGF), three-finger toxin (3FTX), and Kunitz-type serine protease inhibitor (KSPI), each with a transcript level of 0.19%, 0.05%, 0.02%, and below 0.01%, respectively (Table 2). 

The functions of these enigmatic genes are elusive in this species, and again, the classification of some of these minor components as toxins in snake venom is sometimes debatable among scientists. The findings of these components, however, are worth discussing for their potential toxicity and application, such as for drug discovery. Nucleobindin (NCB) is the precursor of nesfatin-1, an endocrine factor associated with epilepsy in mammals [116]. In a proteopeptidome characterization of *Bothrops jararaca* venom, snake venom NCB was speculated to induce excitotoxicity and cause transient disorientation in prey, thus facilitating predation [116]. VEGF is found in some viperid venoms, notably from Russell’s Viper (*Daboia russelii* and *D. siamensis*) [58,117], and its function has been linked to the increment of vascular permeability, the underlying mechanism of capillary leak syndrome [118,119], which is uncommon in *C. rhodostoma* envenoming. The transcriptomic finding of 3FTXs, which are canonical toxins almost exclusive to elapid snakes, is puzzling but not totally unexpected, as some studies have also reported the detection of 3FTx transcripts in the venom glands of crotalid and colubrid snakes [120,121]. Intriguingly, this is the first report of the detection of 3FTX transcripts from a basal Old World pit viper, which is evolutionarily a more primitive species among the advanced venomous snakes. The suggestive presence of these genes across various snake families may imply that these genes probably have a much earlier recruitment that predates the divergence between viperids, elapids, and colubrids, with selection favoring the expansion and neofunctionalization of these genes in the more derived elapid snakes while silencing those in the viperids. The Kunitz-type serine protease inhibitors (KSPI), on the other hand, may serve to inhibit proteolysis by serine proteases in venom. In some viperid venoms, KSPIs such as Ruvikunin and Rusvikunin-II purified from the native Rusvikunin complex of Pakistan’s Russell’s Viper have been shown to exhibit anti-coagulant activity [122]. 

## 3. Conclusions

The present study unravels the complexity and diversity of venom genes in *C. rhodostoma* (Malayan Pit Viper), a basal pit viper species that is endemic to Southeast Asia. The *de novo* assembled venom gland transcriptome uncovers 16 toxin families in which numerous genes with novel sequences were identified. The major toxins determined, along with their differential expression levels, are mostly reflective of the venom proteomic profile of *C. rhodostoma,* notwithstanding inconsistencies that could result from several factors, including limited time points of tissue sampling (which unfortunately ignored the kinetics and time course of venom production), varying mRNA half-lives of toxins post-transcription, and complex regulations of protein translation in the venom gland and subsequent release as secretory proteins into the lumen. The findings derived from a snapshot of the transcriptome profile as such nonetheless provide deep insights into the gene structures and functions of toxins from this unique species, improving our understanding of their relevance in the context of snakebite envenoming. 

The *de novo* sequencing of toxins, many of which have not been fully characterized previously in this species, can enrich snake venom databases and provide information to support further studies in venom evolution, snakebite treatment, antivenom design, and drug discovery. *C. rhodostoma* is relatively basal in the phylogeny of venomous snakes while having evolved into a monotypic species that occupies a distinct ecological niche. Thus, its venom genes are an indispensable reference in the quest for the origin of animal toxins. Insights into the structures of the various toxins will also lead to a better understanding of their protein antigenicity and interaction with antibodies, accelerating the development of antivenom with higher effectiveness. Additionally, the toxin sequences can be further explored as templates for bioactive molecules, thus unleashing their therapeutic potentials toward drug discovery. 

## 4. Materials and Methods

### 4.1. Preparation of C. rhodostoma Venom Gland Tissue

The *C. rhodostoma* specimen was an adult snake collected from Kedah, a northern state in Peninsular Malaya. The venom was milked four days prior to venom gland tissue collection to promote transcription [123]. The venom glands were collected following euthanasia and sectioned into dimensions of 5 × 5 mm. The sectioned tissue was immersed in RNAlater^®^ solution (Ambion, TX, USA) at 4 °C overnight and stored at −80 °C until further use. The study was carried out in line with protocols approved by the Institutional Animal Use and Care Committee (IACUC) of the University of Malaya, Malaysia (Approval code: #2013-11-12/PHAR/R/TCH).

### 4.2. RNA Extraction and mRNA Purification

The venom gland tissue was homogenized in a 1 mL glass homogenizer with TRIzol solution (Invitrogen, Carlsbad, CA, USA). This was followed by the isolation using chloroform and treatment with RNA-free DNAase I (Thermo Fisher Scientific, Waltham, MA, USA) to separate cellular debris and residual DNA. The isolated RNA was then purified with isopropyl alcohol precipitation. Polyadenylated mRNA was subsequently purified with oligo (dT) magnetic beads (Illumina TruSeq Stranded mRNA) (Illumina, San Diego, CA, USA) as per the manufacturer’s instructions. The quality of the purified total RNA was assessed using Agilent 2100 Bioanalyzer (RNA 6000 NanoKit) (Agilent Technologies, Waldbronn, Germany).

Enriched poly(A)+ mRNA isolated from the total venom gland RNA was used for cDNA construction. The isolated mRNA was fragmented into short fragments, which acted as templates for cDNA synthesis [124]. Random hexamer-primer (N6) was used to synthesize the first-stranded cDNA, followed by second-strand cDNA synthesis with the double-stranded cDNA as input materials, using second-strand buffers, dNTPs, RNase H, and DNA polymerase I. From these cDNAs, a paired-end library was synthesized using the Genomic Sample Prep kit (Illumina, San Diego, CA, USA), according to the manufacturer’s instructions. The cDNA fragments generated were purified with QIAquick PCR extraction kit (Qiagen, Valencia, CA, USA) and dissolved in elution buffer for end repair and the addition of poly(A) to aid in the subsequent ligation of Illumina adaptors that contain a single thymine (T) base overhang at the 30 ends. Following the ligation, these cDNA fragments were amplified via polymerase chain reaction (PCR) and electrophoresed on a 1.5–2% TAE (tris base, acetic acid, and EDTA) agarose gel. From the gel, suitable fragments (200–700 bp) were selected as templates for subsequent PCR amplification. Sequencing of the amplified sample library was achieved in a single lane on the Illumina HiSeq™ 2000 platform (Illumina, San Diego, CA, USA) with 100 base-pair, paired-end reads.

### 4.3. Filtration of Raw Sequenced Reads

Sequenced data generated from Illumina HiSeq™ 2000 platform were transformed by base calling into sequence data, called the raw reads, and stored in a FASTQ format. Prior to transcriptome assembly, raw reads were filtered to generate clean reads as part of the quality control process in the pre-analysis stage [125]. This involved the removal of (i) adaptors; (ii) reads with >5% of unknown nucleotides; or (iii) low-quality reads with >20% of low-quality bases (determined as base quality < 10).

### 4.4. De Novo Transcriptome Assembly

The *de novo* transcriptome assembly was performed using a short-reads assembly program, Trinity (version 2.0.6) [126,127]. Three independent software modules, that is, Inchworm, Chrysalis, and Butterfly, comprised the Trinity program and were sequentially applied to process the large volumes of RNA-seq reads. In brief, this was based on the algorithm of *de Bruijn* graph construction, which began by aligning k-mers (k = 25), and reads with a certain length of overlap were joined to form linear contigs. The reads were mapped back onto contigs, and by referring to paired-end reads, contigs from the same transcript as well as the distances between them were determined. The contigs were then partitioned into clusters, each of which carried a complete set of *de Bruijn* graphs (representing the transcriptional complexity at a given gene or locus). The graphs were independently processed to obtain full-length transcripts for alternatively spliced isoforms and to tease apart transcripts that corresponded to paralogous genes. The clean read Q20 percentage, a point of reference for quality control assessment, was obtained as a benchmark for successful *de novo* assembly of the transcriptome. 

### 4.5. Clustering and Functional Annotation of Transcripts

The transcript sequences generated through Trinity were called Unigenes. Unigenes from the transcriptome assembly were further processed for sequence splicing and redundancy removal with TGI clustering tools (TGICL, version 2.1) to acquire non-redundant (NR) transcripts at the longest possible length [128]. The transcripts were then subjected to family clustering, which resulted in two classes of transcripts: (a) clusters, with a prefix CL and the cluster ID behind as contig; (b) singletons, whose ID was simply left with a prefix of Unigene. In each cluster, there were several transcripts with sequence similarities among them of >70%, while the singletons, or “Unigenes” lack overlapping with other fragments at the given stringency. The value of 70% was used to categorize the assembled sequences based on similarity; sequences similar to each other (which may or may not be homologous as having > 90% similarity) were grouped under a cluster comprising various contigs.

Following this, transcript Unigenes were then aligned with BLASTx to the protein database in priority order to NCBI non-redundant database (NR), with a cut-off value of E < 10^−5^. Proteins with the highest ranks in the BLASTx results were referred to determine the coding region sequences of Unigenes, followed by translation into amino acid sequences (using standard codon table). Hence, both nucleotide sequences (50 to 30) and amino acid sequences of the Unigene-coding regions were acquired. To remove redundancy from each cluster, the longest sequence in each cluster was chosen as the transcript; meanwhile, the length of scaffold was extended based on overlapping sequences using Phrap assembler (release 23.0) (http://www.phrap.org, accessed on 20 April 2023). The distributions of the length of contigs, scaffolds, and Unigenes were calculated, and the N50 length (assembly quality indicator) was set at N50 > 500 for assembly success.

### 4.6. Quantifying Transcript Abundance

Clean reads were aligned to Unigene using Bowtie2 [129]. The transcript abundances were calculated using RNA-seq with expectation maximization (RSEM) tool [130]. 

Fragments per kilobase of exon model per million reads mapped (FPKM) were used to determine the transcript abundance for the identified genes [131]. FPKM is the sum of normalized read counts based on gene length and the total number of mapped reads. The data were obtained using the RSEM tool in conjunction with Trinity based on a computational formula:

$$\mathrm{FPKM}\;\mathrm{of}\;\mathrm{gene}\;A=\frac{10^{6}B}{NC/1000}$$

FPKM is the expression of gene A; B is the number of fragments/reads that are aligned to gene A; *N* is the total number of fragments/reads that are aligned to all genes; and *C* is the base number in the coding sequence of gene A. 

### 4.7. Categorization of Transcripts

The *de novo* assembled transcripts were subjected to BLASTx search to obtain the closest-resembling sequences from the NR protein database for further classification based on functional annotations. The transcripts (Unigenes) were then sifted to remove those with an FPKM value of less than 1, followed by categorization into three groups: “toxins,” “non-toxins,” and “unidentified” [29,31]. “Toxin” transcripts were recruited by toxin-related keyword searches against the annotated transcripts. “Non-toxin” and “unidentified” groups contain transcripts of cellular proteins or house-keeping genes and transcripts that could not be identified, respectively. The redundancy of gene expression was determined by dividing the total FPKM of each group by the total number of transcripts in the respective group of transcripts [31]. In the toxin group, the amino acid sequences were used to further validate the toxin identity through the BLASTp suite (Basic Local Alignment Search Tool-Protein) in the UniProt (Universal Protein Resource Knowledgebase) database platform. The transcripts were searched against the Serpentes database (taxid: 8570) and validated based on the lowest E-score value with the highest percentage of sequence similarity (updated as of 29 January 2023).

### 4.8. Sequence Alignment and Analysis

Multiple amino acid sequence alignment was conducted using Jalview software (version 2.10.5; University of Dundee with Dundee Resource for Protein Sequence Analysis and Structure Prediction, Scotland, United Kingdom) [132] and MUSCLE (Multiple Sequence Comparison by Log-Expectation, version 3.8.31, for amino acids) [133] program (accessed on 23 February 2023). Sequences of related species used in multiple amino acid sequence alignment were retrieved from UniProtKB depository (http://www.uniprot.org/, accessed on 14 February 2023). The selection was based on their relevance to the toxins in comparison to elucidate the similarity, variation, and conserved regions of the sequences. Aligned sequences were colored with BLOSUM62 color scheme, where the color intensity reflects the chance of amino acid substitution, i.e., intense purple = low chance of amino acid substitution; white = high chance of amino acid substitution.

### 4.9. Supporting Data

Sequencing data from the venom gland transcriptomics of *C. rhodostoma* was deposited in National Centre for Biotechnology Information (NCBI) Sequence Read Archive (https://submit.ncbi.nlm.nih.gov/subs/sra/) (submitted on 30 September 2019) under SRA accession: PRJNA549256.

### 4.10. Statistical Analyses

The correlation between venom gland transcriptome (present study) and venom proteome data [18] of *C. rhodostoma* was analyzed. Transcripts that co-expressed in both sets of data were selected and assessed statistically (*p*-value < 0.05) with Pearson product-moment correlation coefficient test (SPSS Version 23, IBM, Armonk, NY, USA) to determine the correlativity of data.

## Figures and Tables

**Table 1:** Overview of the NGS (next-generation sequencing) output statistics for the *de novo* assembly of *Calloselasma rhodostoma* venom gland transcriptome.

Parameter	Value
Total raw reads	47,365,132
Total clean reads	46,636,384
Total clean nucleotides (nt)	4,663,638,400
Q20 percentage	97.17%
*N* percentage	0.00%
GC percentage	44.52%
Contigs created	146,916
Total length (nt)	47,039,053
Mean length (nt)	320
N50	632
Unigenes/transcripts assembled	74,445
Total length (nt)	52,612,410
Mean length (nt)	707
N50	1636
Unigene/transcripts assembled (FPKM > 1)	59,348
Unidentified	38,289
Non-toxin	20,962
Toxin	97

**Table 2:** Overview of the families and subtypes of toxin genes in the venom gland transcriptome of *Calloselasma rhodostoma*.

Protein Family/Protein Subtype	Accession No. (Species)	Relative Abundance % (Subtype)
**Snake venom metalloproteinase (SVMP)**		**37.84 (22)**
**P-I SVMP**		**26.51 (4)**
Snake venom metalloproteinase kistomin	P0CB14 (*Calloselasma rhodostoma*)	26.29
Snake venom metalloproteinase BpirMP	P0DL29 (*Bothrops pirajai*)	0.17
Zinc metalloproteinase/disintegrin ussurin	Q7SZD9 (*Gloydius ussuriensis*)	0.03
Group I snake venom metalloproteinase	Q2UXQ3 (*Echis ocellatus*)	0.02
**P-II SVMP**		**7.94 (4)**
Zinc metalloproteinase/disintegrin	P30403 (*Calloselasma rhodostoma*)	7.89
Zinc metalloproteinase/disintegrin ussurin	Q7SZD9 (*Gloydius ussuriensis*)	0.04
Metalloprotease PIIa	V5IWE4 (*Trimeresurus gracilis*)	0.01
Zinc metalloproteinase-disintegrin VMP-II	J9Z332 (*Crotalus adamanteus*)	<0.01
**P-III SVMP**		**3.39 (14)**
Zinc metalloproteinase-disintegrin-like halysase	Q8AWI5 (*Gloydius halys*)	3.32
Metalloprotease P-III	A0A077L6L9 (*Protobothrops elegans*)	0.03
Zinc metalloproteinase-disintegrin-like NaMP	A8QL59 (*Naja atra*)	0.01
Metalloprotease P-III	A0A077L6L9 (*Protobothrops elegans*)	0.01
Flavorase	G1UJB2 (*Protobothrops flavoviridis*)	0.01
Zinc metalloproteinase-disintegrin-like NaMP	A8QL59 (*Naja atra*)	0.01
Metalloprotease P-III 5	A0A077L7M5 (*Protobothrops flavoviridis*)	<0.01
Metalloproteinase	A0A2Z4N9U9 (*Boiga irregularis*)	<0.01
Zinc metalloproteinase-disintegrin-like NaMP	A8QL59 (*Naja atra*)	<0.01
Metalloprotease P-III 5	A0A077L7M5 (*Protobothrops flavoviridis*)	<0.01
Metalloproteinase	A0A2Z4N9U9 (*Boiga irregularis*)	<0.01
Zinc metalloproteinase-disintegrin-like NaMP	A8QL59 (*Naja atra*)	<0.01
Metalloprotease P-III 5	A0A077L7M5 (*Protobothrops flavoviridis*)	<0.01
Zinc metalloproteinase-disintegrin-like NaMP	A8QL59 (*Naja atra*)	<0.01
**Phospholipase A_2_ (PLA_2_)**		**29.02 (15)**
Phospholipase A2	A0A0H3U266 (*Calloselasma rhodostoma*)	16.47
Acidic phospholipase A2 S1E6-c	Q9PVE9 (*Calloselasma rhodostoma*)	5.19
K49 phospholipase A2	A8Y7N3 (*Deinagkistrodon acutus*)	3.62
Acidic phospholipase A2 Ts-A4	Q6H3C7 (*Trimeresurus stejnegeri*)	2.74
Phospholipase A2 homolog	P0DMT1 (*Echis pyramidum leakeyi*)	0.91
Phospholipase A2	A0A0H3U279 (*Ovophis makazayazaya*)	0.06
Phospholipase A2 group IIE	A0A2H4N3A5 (*Bothrops moojeni*)	0.02
Phospholipase A2, group IIE	A0A1J0R065 (*Crotalus atrox*)	0.01
Group 3 secretory phospholipase A2	A0A223PK36 (*Daboia russelii*)	<0.01
Basic phospholipase A2 beta-bungarotoxin A4 chain	Q75S51 (*Bungarus candidus*)	<0.01
Phospholipase A2 isoform 2	H8PG83 (*Parasuta nigriceps*)	<0.01
Group 15 secretory phospholipase A2	A0A223PK35 (*Daboia russelii*)	<0.01
Acidic phospholipase A2 homolog	P29601 (*Bungarus fasciatus*)	<0.01
Acidic phospholipase A2	P00606 (*Bungarus multicinctus*)	<0.01
Group 3 secretory phospholipase A2	A0A223PK36 (*Daboia russelii*)	<0.01
**Bradykinin-potentiating/Angiotensin-converting enzyme inhibitor/C-type natriuretic peptide (BPP/ACEI-CNP)**		**16.30 (3)**
Angiotensin converting enzyme inhibitor and C-type natriuretic peptide	M5A7D0 (*Calloselasma rhodostoma*)	5.77
Angiotensin converting enzyme inhibitor and C-type natriuretic peptide	M5A7D0 (*Calloselasma rhodostoma*)	5.51
Angiotensin converting enzyme inhibitor and C-type natriuretic peptide	M5A7D0 (*Calloselasma rhodostoma*)	5.02
**Snake C-type lectin (CTL)**		**10.01 (7)**
Snaclec rhodocytin subunit beta	Q9I840 (*Calloselasma rhodostoma*)	4.33
C-type lectin	G8FML6 (*Calloselasma rhodostoma*)	3.26
Snaclec rhodocytin subunit alpha	Q9I841 (*Calloselasma rhodostoma*)	1.76
Snaclec rhodocetin subunit delta	D2YW40 (*Calloselasma rhodostoma*)	0.37
C-type lectin beta subunit	T2HPS7 (*Protobothrops flavoviridis*)	0.27
Lectoxin-Enh9	A7XQ58 (*Pseudoferania polylepis*)	0.01
C-type lectin 3	A0A346CLX6 (*Ahaetulla prasina*)	0.01
**Snake venom serine proteinase (SVSP)**		**2.81 (14)**
Thrombin-like enzyme ancrod	P26324 (*Calloselasma rhodostoma*)	1.92
Snake venom serine protease 3	O13058 (*Protobothrops flavoviridis*)	0.20
Snake venom serine protease ussurin	Q8UUJ2 (*Gloydius ussuriensis*)	0.19
Snake venom serine protease gussurobin	Q8UVX1 (*Gloydius ussuriensis*)	0.14
Venom thrombin-like enzyme	Q90Z47 (*Deinagkistrodon acutus*)	0.12
Thrombin-like enzyme	Q98TT5 (*Deinagkistrodon acutus*)	0.08
Thrombin-like enzyme stejnobin	Q8AY81 (*Trimeresurus stejnegeri*)	0.08
Snake venom serine protease 3	O13063 (*Trimeresurus gramineus*)	0.02
Venom plasminogen activator GPV-PA	P0DJF5 (*Trimeresurus albolabris*)	0.02
Thrombin-like enzyme ancrod-2	P47797 (*Calloselasma rhodostoma*)	0.02
Serine protease 3	A0A286S0D3 (*Gloydius intermedius*)	0.01
Thrombin-like enzyme kangshuanmei	P85109 (*Gloydius brevicaudus*)	0.01
Serine proteinase isoform 7	B0VXT9 (*Sistrurus catenatus edwardsii*)	<0.01
Thrombin-like protein DAV-WY	B3V4Z6 (*Deinagkistrodon acutus*)	<0.01
**L-amino acid oxidase (LAAO)**		**2.25 (1)**
L-amino-acid oxidase	P81382 (*Calloselasma rhodostoma*)	2.25
**Cysteine-rich secretory protein (CRiSP)**		**0.90 (2)**
Cysteine-rich secretory protein LCCL domain-containing 2	V8NV17 (*Ophiophagus hannah*)	0.90
Cysteine-rich seceretory protein Bc-CRPa	F2Q6G3 (*Bungarus candidus*)	<0.01
**5′-Nucleotidase (5′NT)**		**0.28 (5)**
Snake venom 5’-nucleotidase	F8S0Z7 (*Crotalus adamanteus*)	0.27
5’-nucleotidase	A6MFL8 (*Demansia vestigiata*)	<0.01
5’-nucleotidase	A6MFL8 (*Demansia vestigiata*)	<0.01
5’-nucleotidase	A6MFL8 (*Demansia vestigiata*)	<0.01
5’-nucleotidase 1	A0A346CLX4 (*Borikenophis portoricensis*)	<0.01
**Phospholipase B (PLB)**		**0.25 (4)**
Phospholipase B-like	A0A2H4N395 (*Bothrops moojeni*)	0.25
Phospholipase B1, membrane-associated	V8NN21 (*Ophiophagus hannah*)	<0.01
Phospholipase B-like	V8NLQ9 (*Ophiophagus hannah*)	<0.01
Phospholipase B-like	V8NLQ9 (*Ophiophagus hannah*)	<0.01
**Nucleobindin (NLB)**		**0.19 (1)**
Nucleobindin-1	V8P8E3 (*Ophiophagus hannah*)	0.19
**Nerve growth factor**		**0.07 (1)**
Nerve growth factor	B1Q3K2 (*Protobothrops flavoviridis*)	0.07
**Snake venom vascular endothelial growth factor (VEGF)**		**0.05 (1)**
Snake venom vascular endothelial growth factor toxin	P67862 (*Protobothrops flavoviridis*)	0.05
**Three-finger toxin (3FTX)**		**0.02 (9)**
Alpha-bungarotoxin isoform A31	P60615 (*Bungarus multicinctus*)	0.01
Neurotoxin-like protein pMD18-NTL1/2/4/5	Q7ZT13 (*Bungarus multicinctus*)	<0.01
Muscarinic toxin BM14	Q8JFX7 (*Bungarus multicinctus*)	<0.01
Kappa-3-bungarotoxin	P15817 (*Bungarus multicinctus*)	<0.01
Gamma-bungarotoxin	Q9YGJ0 (*Bungarus multicinctus*)	<0.01
Three finger toxin 1	A5X2W6 (*Sistrurus catenatus edwardsii*)	<0.01
Short neurotoxin homolog NTL4	Q9YGI8 (*Bungarus multicinctus*)	<0.01
Three finger toxin 2	A5X2W7 (*Sistrurus catenatus edwardsii*)	<0.01
Putative three finger toxin	F5CPD4 (*Micrurus altirostris*)	<0.01
**Aminopeptidase A**		**0.01 (1)**
Aminopeptidase	T2HQN1 (*Ovophis okinavensis*)	0.01
**Phosphodiesterase (PDE)**		**0.01 (5)**
Venom phosphodiesterase 1	J3SEZ3 (*Crotalus adamanteus*)	<0.01
Venom phosphodiesterase 1	J3SEZ3 (*Crotalus adamanteus*)	<0.01
Venom phosphodiesterase 2	J3SBP3 (*Crotalus adamanteus*)	<0.01
Venom phosphodiesterase 1	J3SEZ3 (*Crotalus adamanteus*)	<0.01
Venom phosphodiesterase 2	J3SBP3 (*Crotalus adamanteus*)	<0.01
**Kunitz-type serine proteinase inhibitor (KSPI)**		**<0.01 (1)**
Kunitz-type serine protease inhibitor homolog beta-bungarotoxin B2a chain	Q8AY45 (*Bungarus candidus*)	<0.01

**Table 3:** Full-length toxin transcripts derived from the venom gland transcriptome of *Calloselasma rhodostoma*.

Protein family/Protein ID		Annotated Accession	Species	Amino Acid Chain	Mature Chain of Accession ID	Coverage (Mature Chain)	Coverage Percentage (%)
**Snake venom metalloproteinase (SVMP)**							
Cr-SVMP01	Snake venom metalloproteinase kistomin	P0CB14	*Calloselasma rhodostoma*	417	417	1–417	100
Cr-SVMP05	Zinc metalloproteinase/disintegrin	P30403	*Calloselasma rhodostoma*	478	478	1–478	100
**Phospholipase A_2_ (PLA_2_)**							
Cr-PLA04	Acidic phospholipase A2 Ts-A4	Q6H3C7	*Trimeresurus stejnegeri*	139	139	1–139	100
Cr-PLA10	Basic phospholipase A2 beta-bungarotoxin A4 chain	Q75S51	*Bungarus candidus*	147	147	1–147	100
Cr-PLA11	Phospholipase A2 isoform 2	H8PG83	*Parasuta nigriceps*	136	136	1–136	100
Cr-PLA12	Group 15 secretory phospholipase A2	A0A223PK35	*Daboia russelii*	362	393	1–341	92
**Snake C-type lectin (Snaclec)**							
Cr-CTL01	Snaclec rhodocytin subunit beta	Q9I840	*Calloselasma rhodostoma*	146	146	1–146	100
Cr-CTL02	C-type lectin	G8FML6	*Agkistrodon piscivorus* *leucostoma*	157	158	1–158	99
Cr-CTL03	Snaclec rhodocytin subunit alpha	Q9I841	*Calloselasma rhodostoma*	136	136	1–136	100
Cr-CTL04	Snaclec rhodocetin subunit beta	P81398	*Calloselasma rhodostoma*	129	129	1–129	100
Cr-CTL05	Snaclec rhodocetin subunit delta	D2YW40	*Calloselasma rhodostoma*	150	150	1–150	100
**Snake venom serine proteinase (SVSP)**							
Cr-SSP01	Thrombin-like enzyme ancrod	P26324	*Calloselasma rhodostoma*	234	234	1–234	100
Cr-SSP03	Snake venom serine protease ussurin	Q8UUJ2	*Gloydius ussuriensis*	224	236	13–236	95
**L-amino acid oxidase (LAAO)**							
Cr-LAO01	L-amino-acid oxidase	P81382	*Calloselasma rhodostoma*	516	516	1–516	100
**Cysteine-rich** **venom protein (CRiSP)**							
Cr-CRP01	Cysteine-rich secretory protein LCCL domain-containing 2	V8NV17	*Ophiphagus Hannah*	495	472	1–472	100
**5′-Nucleotidase (5′NT)**							
Cr-NUC01	Snake venom 5’-nucleotidase	F8S0Z7	*Crotalus adamanteus*	588	588	1–588	100
**Phospholipase B (PLB)**							
Cr-PLB01	Phospholipase B-like	A0A2H4N395	*Bothrops moojeni*	553	558	1–553	99
Cr-PLB03	Phospholipase B-like	V8NLQ9	*Ophiophagus Hannah*	321	300	87–299	100
**Nucleobindin (NLB)**							
Cr-NLB01	Nucleobindin-1	V8P8E3	*Ophiophagus Hannah*	452	397	22–372	100
**Nerve growth factor (NGF)**							
Cr-NGF01	Nerve growth factor	B1Q3K2	*Protobothrops flavoviridis*	237	241	1–237	98
**Snake venom vascular endothelial growth factor (VEGF)**							
Cr-VGF01	Snake venom vascular endothelial growth factor toxin	P67862	*Protobothrops flavoviridis*	145	146	1–145	99
**Three finger toxin (3FTX)**							
Cr-FTX01	Alpha-bungarotoxin isoform A31	P60615	*Bungarus multicinctus*	95	95	1–95	100
Cr-FTX02	Neurotoxin-like protein pMD18-NTL1/2/4/5	Q7ZT13	*Bungarus multicinctus*	86	86	1–86	100
Cr-FTX03	Muscarinic toxin BM14	Q8JFX7	*Bungarus multicinctus*	97	103	7–103	94
**Aminopeptidase A**							
Cr-APP01	Aminopeptidase	T2HQN1	*Ovophis okinavensis*	953	953	1–953	100
**Phosphodiesterase (PDE)**							
Cr-PDE02	Venom phosphodiesterase 1	J3SEZ3	*Crotalus adamanteus*	844	851	6–849	99
Cr-PDE03	Venom phosphodiesterase 2	J3SBP3	*Crotalus adamanteus*	808	810	1–808	99
Cr-PDE04	Venom phosphodiesterase 1	J3SEZ3	*Crotalus adamanteus*	849	851	1–849	99
Cr-PDE05	Venom phosphodiesterase 2	J3SBP3	*Crotalus adamanteus*	803	810	6–808	99
**Kunitz-type serine proteinase inhibitor (KSPI)**							
Cr-KUN01	Kunitz-type serine protease inhibitor homolog beta-bungarotoxin B2a chain	Q8AY45	*Bungarus candidus*	84	85	2–85	99

## Data Availability

Data are contained within the article and Supplementary Materials. Access to sequencing data is described in Section 4.9.

## Supplementary Materials

The following supporting information can be downloaded at: https://www.mdpi.com/article/10.3390/toxins15050315/s1, Supplementary Files S1 and S2.
