# Supplementary material for: De Novo Venom Gland Transcriptome Assembly and Characterization for Calloselasma rhodostoma (Kuhl, 1824), the Malayan Pit Viper from Malaysia: Unravelling Toxin Gene Diversity in a Medically Important Basal Crotaline
Source: Toxins (Basel). 2023 Apr 29;15(5):315. doi: 10.3390/toxins15050315 (PMC10222492; doi:10.3390/toxins15050315)
Supplement: Supplementary file 1 [file toxins-15-00315-s001.zip › Supplementary File S2.pdf]

Supplementary File for low abundance transcripts

Accession		Amino acid sequence	
V8NV17 CRiSP	1	MAKNPSIPWLIPPTLFLFLTHGIHCFFLPNATHLENILNQYQDGQPHSRAKRSISRSDQD	60
LCCL domain_Oh	61	EILMLHNKLRGEVYPSASNMEYM-------------------------NSTHFPAGQRLF	120
	121	HPLHRSPSFHVQSWYDEVKDYTYPYPHECNPWCPDRCTGPMCTHYTQMVWATTNKIGCAI	180
	181	NVCKRIDVWGEVWENAVYLVCNYSPKGNWIGEAPYKNGRSCSECPPSYGGGCRANLCYKE	240
	241	KVVEKPETDRANEVELQKVPVRIQPKSVKPTKEKKPTEVNYMTQVIKCDTKMRDTCRGST	300
	301	CNRYLCPAGCLNSKAKVFGFPKYESASSICRAAIHSGVLDNRGGLVDITRKGRSDFFVMS	360
	361	VRNGVQTFSKYKPSNGFAVSKVTVQTLDCYTTVEELCPFKKPTTHCPRRLKSSSRKAKRE	420
	421	SHNPSMPAMEQMCKNSSICKAAVHAGVIGDSSGGYVDVMPVDKKKVYNGSLRNGIRSESL	480
	481	KTPREGKAFRIFAVRQ	496
Cr-CRP01	1	MAKNPTVPWLVAPSLFLFLTHGIQCFFLPSATHLERILNQYQDGQPHSRGKRSISRDDRD	60
	61	EILTLHNKLRGEVYPSASNMEYMRWDEELERSAESWAQQCIWDHGPASLLLSIGQNLAVH	120
	121	WGRHRSPGYHVQSWYDEVKDYTYPYPHECNPRCPDRCRGPVCTHYTQMVWATTKNMGCAI	180
	181	NVCKRMVVWGEVWENAVYLVCNYSPKGNWIGEAPYKNGRPCSECPPSYGGGCQANLCYKE	240
	241	KAVEKSETDRANEVEIQKVPDRIQPKSVKPAKEKKPVEVTYMTQAIKCDTKMRDTCRGST	300
	301	CNRYLCPAGCLNSKAKVFGFPNYESASSICRAAIHSGVLDNRGGLVDITRKGRSDFFVMS	360
	361	VRNGIRTLSKHKPSNGFAVLKVIVQTLDCYTTVEELCPFKKPTTHCPRSYCPAYCKNE-P	420
	421	NHWATVYGTNIYADNSSICKAAVHAGVIGDSSGGYVDVMPVDKKKVYNGSLRNGIRSESL	480
	481	KTPREGKAFRIFAVRQ	496

Multiple amino acid sequence alignment of Cr-CPR01 (Putative cysteine-rich secretory protein (CRiSP)) identified in *Calloselasma rhodostoma* venom gland transcriptome in comparison to selected CRiSP retrieved from UniProt KB database, Cysteine-rich secretory protein LCCL domain-containing 2, UniProt ID: V8NV17. Abbreviation: Oh – *Ophiophagus hannah*

Accession		Amino acid sequence	
V5NTD 5’NUC Ca	1	MQTPKRRRGAQGCPRSSPS--PPLLLLVRAVWFCAALSVAAGSFELTILHTNDVHARVEQ	60
	61	TSRDSGKCTGQDCYGGVARRATKIRELRAKHRHVLLLDAGDQYQGTVWFNFFKGREVVKF	120
	121	MNSLRYDAMALGNHEFDNGLAGLLDPLLKHANFPILSANIRPKGSIASNISGYILPYKII	180
	181	NVGSEKVGIIGYTTKETPVLSNPGPYLEFRDEVEELQNHANKLTTLGVNKIIALGHSGFS	240
	241	EDQRIARKVKGVDVVVGGHTNTFLYTGSPPSTEVAAGNYPFMVQSDDGRQVPVVQAYAFG	300
	301	KYLGYLNVIFDDKGNVIKSSGNPILLNKDISEDQDIKAEVNKMKIQLHNYSSQEIGKTIV	360
	361	YLNGTTQACRFHECNLGNLICDAVIYNNVRHPDDNEWNHVSMCIVNGGGIRSPIDERTNN	420
	421	GTITLEELTAVLPFGGTFDLLQIKGSALKQAFEHSVHRHGEGMGELLQVSGIKVVYDLSR	480
	481	KPGSRVLSLNVLCTECRVPTYVPLEKEKTYKLLLPSFLAAGGDGYHMLKGDSSNHSSGNL	540
	541	DISIVGDYIKRMGKVFPAVEGRMIFSAGTLFQAQLFLTWGLCVSLLYFIL	590
Cr-NUC01	1	MQTPKR--GTPGCPPSCPSPPPPPPLLVGAVWFCALLSLAAASFELTLLHTNDVHARVEQ	60
	61	TSRDSGKCTGQECYGGVARRATKIRELRAKHRHVLLLDAGDQYQGTIWFNFFKGREVVTF	120
	121	MNRLRYDAMALGNHEFDNGLAGVLDPLLKYASFPILSANIIPKGPIASNISGYILPYKII	180
	181	NVGSEKVGIIGYTTKETPVLSNPGPYLEFRDEVEELQKHVNKLTTLGVNKIIALGHSGFL	240
	241	EDQRIAQKVKGVDVVIGGHTNTFLYTGTPPSTEVAAGNYPFMVQSDDGRQVPVVQAYAFG	300
	301	KYLGYLNVIFDDKGNVIKASGNPILLNKDIPEDQDVKTEVNKMKIQLHNYSSQEIGKTIV	360
	361	YLNGTTHACRFHECNLGNLICDAVIYDNVRHPDDNEWNHVSMCIINGGGIRSPIDERANN	420
	421	GTITLEELTAVLPFGGTFDLLQVKGDALKQAFEHSVHRHGQGTGELLQTSGIKVVYDLSR	480
	481	RPGMRVVSLKVLCTECRVPTYVPLETGKTYKLLLPSFLASGGDGYHMLKGDASNHSSGNL	540
	541	DISIVGDYIKRMGKVFPAVEGRMIFSAGTLFQAQLFLTWALCISLLYFIL	590

Multiple amino acid sequence alignment of Cr-NUC01 (Putative 5’-nucleotidase (5’NT)) identified in *Calloselasma rhodostoma* venom gland transcriptome in comparison to selected 5’NUC retrieved from UniProt KB database, Snake venom 5'-nucleotidase, UniProt ID: F8S0Z7. Abbreviation: Ca – *Crotalus adamanteus*

Accession		Amino acid sequence	
A0A2H4N395 PLB-like Bm	1	MIRFGNPSSS----DKRRQRCRSWCWGG-LLLLWAVAETRADIHYATVYWLEAEKSFQIK	60
	61	DVLDKNGDAYGYYNDTIQSTGWGILEIKAGYGNQPISNEILMYAAGFLEGYLTASHMSDH	120
	121	FANLFPLMIKNVIIEQKVKDFIQKQDEWTRQQIKNNKDDPFWRNAGYVIAQLDGLYMGNV	180
	181	EWAKRQKRTPLTDFEISFLNAIGDLLDLIPALQSTLRKSDFRSMPDVPRIYQWDMGHCSA	240
	241	LIKVLPGYENIYFAHSSWFTYAATLRIYKHLDFKITDPQTKTGRASFSSYPGFLSSLDDF	300
	301	YILGSGLTMLQTTNSVLNLSLLKKVVPESLFAWERVRIANMMADSGKTWAETFEKQNSGT	360
	361	YNNQYMILDTKKIKLQRSLEDGTLYIIEQVPKLVKYSDQTKVLRNGYWPSYNIPFDKEIY	420
	421	NMSGYGEYVQRHGLEFSYEMAPRAKIFRRDQGKITDMESMKSIMRYNNYKEDPYAKRNPC	480
	481	NTICCRQDLDRRTPVPAGCYDSKVADISMAAKFTAYAISGPTVEKGLPVFSWVNFNKTKH	540
	541	QGLPESYNFDFVTMKPVL	558
Cr-PLB01	1	MIRLGHPSSSSSSWEKRRQRCRSWSWGGLLLLLWAVAETRADIHYATVYWLEAEKSFQIK	60
	61	DVLDKNGNAYGYYNDTIQSTGWGILEIKAGYGNQPISNEILMYAAGFLEGYLTASHMSDH	120
	121	FANLYPLMIKNVIIEQKVKDFIQKQDKWTRQQIKNNKDDPFWRNAGYVIAQLDGLYMGNV	180
	181	EWAKRQKKTPLTIFKISFLNAVGDLLDLIPALYSNLRKSDVRSMPDVPRIYQWDMGHCSA	240
	241	LIKVLPGYENIYFAHSSWFTYAATLRIYKHLDFKITDPQTKTGRASFSSYPGFLVSLDDF	300
	301	YILGNGMVLLQTTNSVFNLSLLKKVVPESLFAWERVRIANMMADSGKTWAETFEKQNSGT	360
	361	YNNQYMIIDTKKIKLRRSLEDGTLYIVEQVPKLVKYSDQTKVLRNGYWSSYNIPFDKVIY	420
	421	NISGYGEYVQRFGLEFSYEMAPRAKIFRRDQGKVTDMESMKSIMRYNNYKEDPYAKHNPC	480
	481	NTICCRQDLDRRSPVPAGCYDSKVTDISMAAKFTAYAINGPPVEKGLPVFSWIDFNKTKH	540
	541	QGLPESYNFDFVIMKPVL	558

Multiple amino acid sequence alignment of Cr-PLB01 (Putative phospholipase B (PLB)) identified in *Calloselasma rhodostoma* venom gland transcriptome in comparison to selected PLB retrieved from UniProt KB database, Phospholipase B-like, UniProt ID: A0A2H4N395. Abbreviation: Bm – *Bothrops moojeni*

Accession		Amino acid sequence	
V8P8E3 NLB Oh	1	--	60
	61	----MCWKPMGISGKNYKQPMPRTSSKELDFVGHSVRTKLDELKRQEVSRLRMLLKAKMD	120
	121	ATMEQDVQVDHLALLKQFEHLDSQNQHTFEARDLELLIKTATKDLENYDAAHHEEFKRYE	180
	181	MMKEHERREYLKSLDEEKRREEEAHFEELKKKHKEHPKVNVP—TPTVMGCWMSRNWRHFS	240
	241	LRSGGVEFREMQFEHSRGAPAKEV---FFLQLEKVYDPRNEEDDMLEMEEERLRMREHVM	300
	301	KNVDLNKDRLVTLDEFVKSTQRKEFNEADGWETVEETQIYSEAELQRFEAELKAQEEELS	360
	361	RRAEQLHQEHKELQQRQVELDAQKKEYQQVVLQMEQRKSQQLEQAPQVGPGGELKFQAQP	420
	421	PHAAPADHASPPESQPGAPGAPEHDSQKDQTEALQNQAHPEVAVQ	480
Cr-NLB01	1	QIRWRTTLLQQCVLLLTYIVIILEAVPIDVDKTKVKEEEKDSATVENPDTGLYYDAYLRQ	60
	61	VIEVLETDKHFREKLQTADIEQIKSGKLSKELDLVGHKVRTKLDELKRQEVARLRMLIKA	120
	121	KIDAYQDSGVDHQALLKQFGHLNHNNPHTFEVKDLDMLIKTATHDLENYDNERHEEFKKY	180
	181	EMMKEHERREYLKTLDEEKRHQEEAKYEDMKKKHNDHPKVNHPGSKDQLKEVWEEADGLD	240
	241	PNDFDPKTFFKLHDVNADGFLDEQELEALFTRELEKVYDPRNEEDDMVEMEEERLRMREH	300
	301	VMNEVDINKDRLVTLEEFMRATEKREFLEPENWETLDQQQVFTEDDLKEFESHIVQKEDE	360
	361	LQKQALELQKKREELQQQDFLQAQKQELEMAVKQMEQKKLQQGH—PPSGPGGELKFQ--	420
	421	-PSVSQLD—GNVQNHPVAPGNIPVQEAAARSDHVQSH--------	480

Multiple amino acid sequence alignment of Cr-NLB01 (Nucleobindin (NLB)) identified in *Calloselasma rhodostoma* venom gland transcriptome in comparison to selected NLB retrieved from UniProt KB database, Nucleobindin-1, UniProt ID: V8P8E3. Abbreviation: Oh – *Ophiophagus hannah*

Accession		Amino acid sequence	
B1Q3K2 NGF Pf	1	MSMLCYTLIIAFLIGIWAAPKSEDNVPLGSPATSDLSDTSCAKTHEALKTSRNTDQHYPA	60
	61	PKKAEDQEFGSAANIIVDPKLFQKRRFQSPRVLFSTQPPPLSRDEQSVDNANSLNRNIRS	120
	121	KREDHPVHNRGEYSVCDSVSVWVANKTTATDIRGNLVTVMVDVNLNNNVYKQYFFETKCR	180
	181	NPNPVPTGCRGIDSRHWNSYCTTTHTFVRALTMEGNQASWRFIRIDTACVCVISRKNENF	240
	241	G	
Cr-NGF01	1	MSMLCYTLIIAFLIGIWAAPKSEDNVPLGSPATSDLSDNSCAKTHEALKTSRNTDQHYPA	60
	61	PKKAEDQEFGSAANIIVDPKLFQRRRFQSPRVLFSTQPPPLSRDEQSVDNANSLNRNIRA	120
	121	KREDHPVHNRGEYSVCDSVNVWVANKTTATDIRGNVVTVMVDINLNNNAYKQYFFETKCR	180
	181	NPNPVPTGCRGIDARHWNSYCTTTHTFVRALTMEGNQASWRFIRIDTACVCVISRKN---	237
	241	-	

Multiple amino acid sequence alignment of Cr-NGF01 (Putative nerve growth factor (NGF)) identified in *Calloselasma rhodostoma* venom gland transcriptome in comparison to selected NGF retrieved from UniProt KB database, Nerve growth factor, UniProt ID: B1Q3K2. Abbreviation: Pf – *Protobothrops flavoviridis*

Accession		Amino acid sequence	
P67862 VEGF Pf	1	MAAYLLAVAILFCIQGWPSGTVQGQVMPFMEVYSRSACQTRETLVPILKEYPDEVSHLFK	60
	61	PSCVPVLRCGGCCSDESLTCTATGKHSVGREIMRVDPHKGTSKMEVMQFKEHTACECRPR	120
	121	SPGDVNNGKDKRNPEEGGPRARFPFV	180
Cr-NLB01	1	MAAYLLAVAILFCIQGWPSGTVQGQVMPFMEVYSRSACQTRETLVPILKEYPDEVSHLFK	60
	61	PSCVPVLRCGGCCSDESLTCTATGKHSVGREIMRVDPHKGTSKMEVMQFKEHTACECRPR	120
	121	SPGDVNNGKDKRNPEEGGPRARFPFV	180

Multiple amino acid sequence alignment of Cr-VGF01 (Snake venom vascular endothelial growth factor (VEGF)) identified in *Calloselasma rhodostoma* venom gland transcriptome in comparison to selected VGF retrieved from UniProt KB database, Snake venom vascular endothelial growth factor toxin, UniProt ID: P67862. Abbreviation: Pf – *Protobothrops flavoviridis*

Accession		Amino acid sequence	
P60615 Alpha	1	MKTLLLTLVVVTIVCLDLGYTIVCHTTATSPISAVTCPPGENLCYRKMWCDAFCSSRGKV	60
bungarotoxin Bm	61	VELGCAATCPSKKPYEEVTCCSTDKCNPHPKQRPG	120
Cr-FTX01	1	MKTLLLTLVVVTIVCLDLGYTIVCHTTATSPISAVTCPPGENLCYRKMWCDAFCSSRGKV	60
	61	VELGCAATCPSKKPYEEVTCCSTDKCNPHPKQRPG	120
Q7ZT13 NTX-like	1	MKTLLLTLVVLTIACLDLGYTKTCFNDDLTNPKTTELCRHSMYFCFKNSWIAGGVERIER	60
Bm	61	GCSLTCPDIKYNGKYIYCCTRDNCNA	120
Cr-FTX02	1	MKTLLLTLVVLTIACLDLGYTKTCFNDDLTNPKTTELCRHSMYFCFKNSWIAGGVERIER	60
	61	GCSLTCPDIKYNGKYIYCCTRDNCNA	120
Q8JFX7 MTX Bm	1	MKTLLLTLVVVTIICLDLGYTEMCNMCVRPYPFMSSCCPEGQDRCYKSYWVNENGKQKKYHG	60
	61	KYPVILERGCVTACTGPGSGSIYNLYTCCPTNRCGSSSTSG	120
Cr-FTX03	1	------TLVVVTIICLDLGYTEMCNMCVRPYPFMSSCCPEGQDRCYKSYWVNENGKQKKYHG	60
	61	KYPVILERGCVTACTGPGSGSIYNLYTCCPTNRCGSSSTSG	120
P15817 Kappa	1	MKTLLLSLVVVTIVCLDLGYTRTCLISPSSTPQTCPNGQDICFRKAQCDNFCHSRGPVIEQG	60
bungarotoxin Bm	61	CVATCPQFRSNYRSLLCCRTDNCNH	120
Cr-FTX04	1	--------------CLDLGYTRTCLISPSSTPQTCPNGQDICFRKAQCDNFCAMRGPVIEQG	60
	61	CVATCPEFRSNYISLLCCRTDNCN-	120
Q9YGJ0 Gamma	1	MKTLLLTLVVVTIVCLDLGYTMQCKTCSFYTCPNSETCPDGKNICVKRSWTAVRGDGPKREI	60
bungarotoxin Bm	61	RRECAATCPPSKLGLTVFCCTTDNCNH	120
Cr-FTX05	1	-------------VCLDLGYTLKCKTCPFYNCPNSETCPGGKNICVKRSWTAVRGDGPKHEI	60
	61	RRECAATCPPKKPGLTVFCCTTDNCNH	120
A5X2W6 3FTX Sce	1	MKTLLVILGVVAFVYLDSGYSLECYSCNMTFSILPLKLCRSVMCPEGLDQCYINKTLFPV	60
	61	LKIEKGCTTNCTQTWTDKCCETNKCNII	120
Cr-FTX06	1	---------VAMFYFNHLGYSIQCYQCNNPCSRRVL------MTCPESLRQCYTNKTDTS	60
	61	GRPAGKGCTMNCIENEQIKCCNTSRCN--	120
Q9YGI8 SNTX Bm	1	MKTLLLTLVVVTIICLDLGYTRKCLIKYSQANESSKTCPSGQLLCLKKWEIGNPSGKEVK	60
	61	RGCVATCPKPKKNEIIQCCAKDKCNK	120
Cr-FTX07	1	------TLVVLTIVCLDLGYTRKCLIKYSQANESSKTCPSGQLLCLKKWEIGNPSGKEVK	60
	61	RGCVATC-------------------	120
A5X2W7 3FTX Sce	1	MKTLLVTVGVVAFVYLEPGYSLICEACNLPNCDFLPARPCPKGFNQCYKKWNLIGLSVMNIE	60
	61	RGCTANCTPNAQTKCCNTNLCNA	120
Cr-FTX08	1	------------------GYSIQCYQCNNP-CSRRVLMTCPESLRQCYT--NKTDTSGRPAG	60
	61	KGCTMNCIENEQIKCCNTSRCN-	120
F5CPD4 3FTX Ma	1	MKALLFALFLVAFLFKDPVKSMQCYKCGVSGCHLKITCSEDEKFCYKRYNKISFIRTYGCAK	60
	61	TCTEENNWTASVYCCTTNLCNT	120
Cr-FTX09	1	-KALLSALLLVAFLFKDPVKSMECYRCGVSGCHLKITCSAEETFCYKWLNKIS---------	60
	61	----------------------	120

Multiple amino acid sequence alignment of Cr-FTX01, Cr-FTX02, Cr-FTX03, Cr-FTX04, Cr-FTX05, Cr-FTX06, Cr-FTX07, Cr-FTX08, Cr-FTX09 (Three-finger toxin (3FTX)) identified in *Calloselasma rhodostoma* venom gland transcriptome in comparison to selected 3FTX retrieved from UniProt KB database, Alpha bungarotoxin, UniProt ID: P60615; Neurotoxin-like protein, UniProt ID: Q7ZT13; Muscarinic toxin, UniProt ID: Q8JFX7; Kappa bungarotoxin, UniProt ID: P15817; Gamma bungarotoxin, UniProt ID: Q9YGJ0; 3FTX, UniProt ID: A5X2W6, A5X2W7 and F5CPD4; Short neurotoxin, UniProt ID: Q9YGI8. Abbreviation: NTX – Neurotoxin; MTX – Muscarinic toxin; 3FTX – Three-finger toxin; SNTX – Short neurotoxin; Bm – *Bungarus multicinctus*; Sce – *Sistrurus catenatus edwardsii*; Ma – *Micrurus altirostris*

Accession		Amino acid sequence	
T2HQN1 APP Oo	1	MQGMDIEDKSSKMHCMKGKHVAIICGVVIAVGLILGLGLGLGLKPEACNPPEDNGQVSTK	60
	61	PPTSSTPDVTNPSGSSVFCSAKNDENGAWTNFRLPNYVHPVHYDLDLTPEMEAEVYTGMV	120
	121	NISIRLEEQTTRHLWLHLRETKITEMPQLRISSGQVIEIKRCFGYEPQEYVVIEAEEDLR	180
	181	PGNYFLSMKFKGYLNGSLVGFYSTTYGENGKTKYIAATDHEPTDARKSFPCFDEPNKKAT	240
	241	YTISITHEQDYEAISNMPVEKTISLDNKWTKTIFKKSVPMSTYLVAWAVHQFKYEERISA	300
	301	RGIPLRVYAQPQQINTAIYAANVTKVVFDYFENYFNMNYSLPKLDKIAIPDFGTGAMENW	360
	361	GLITYRETNLLYDSRESAASNKQRVAAVVAHELVHQWFGNIVTMDWWDDLWLNEGFASFF	420
	421	EFMGVNATEEKWQMLDQILIDDLLPVLKDDSLVSSHPITVNVSSPDEITSVFDGISYSKG	480
	481	ASILRMLEDWISPDHFRAGCQKYLKDHYFKNAKTDDFWKAMQEVSGKPVREVMDTWTRQM	540
	541	GYPVLKVALNSTVTQQRFLLDPKADPSKPFSQFSYKWNIPVKWKEGNTSSITFYNKSEVA	600
	601	GITITRPSNLPPDSFLKVNKDHVGFYRVNYEPQVWRTLADIMMKDHQNFNLADRAGFIDD	660
	661	AFALARAGLLKYADALNLTRYLQNETEYIPWQRAVVAVSYIGQMVEDDKALYPKFQRYFG	720
	721	SLVKPIASELKWENDEDHIKSLLRTTVLEFACNMDDPEALGNASLLFKNWTSGISLDVNL	780
	781	RLLVYRFGMQHSGDEEAWNYMFEKYRTATLAQEKEKLLYGLASVKNITLLNRFLNCIKNT	840
	841	TLIRSQDVFTVLRYISFNSYGKTMAWDWVRLNWEYLVKRYTLNDRNLGRLISRISGTFNT	900
	901	ELQLWQMENFFERYPDAGAGEASRKQALETTKSNIEWLKQYRDDVATWLENSE-----	960
Cr-APP01	1	MQGMDIEDKSSKMPCMKGKHVAIICGVVIAVGLILGLGLGLGLKPEACSPPEDNGQVSTQ	60
	61	PPTSSTPDVTSASGSSVFCSAQNDEIGAWSNFRLPSYVLPVHYDLDLTPEMEAEVYTGMV	120
	121	NISIRLEKLTTKHLWLHLRETKITEMPQLRTSSGQVIEIKRCFGYEPQEYVVVEAEEELR	180
	181	PGNYFLSMKFKGYLNGSLVGFYSTTYGENGKTKYIAATDHEPTDARKSFPCFDEPNKKAT	240
	241	YTISITHERDYEAISNMPVEKTISLDNKWTKTIFKKSVPMSTYLVAWAVHQFKYEERISS	300
	301	RGIPLRVYAQPQQINTAIYAANVTKVVFDYFENYFNMNYSLPKLDEIAIPDFGTGAMENW	360
	361	GLITYRETNLLYDSQESAASNKQRVAAVVAHEVVHQWFGNIVTMDWWDDLWLNEGFASFF	420
	421	EFMGVNATEEKWQMLDQILIDDLLPVLKDDSLVSSHAITVNVSSPDEITSVFDGISYSKG	480
	481	ASILRMLEDWISPDQFKAGCQKYLTDHYFKNAKTDDFWKAMQVVSGKPVKEVMDTWTRQM	540
	541	GYPVLKVALNSTITQQRFLLDPKADPSQPFSQFSYKWNIPVKWKEGNTSSITFYNKSELA	600
	601	GITITRPSDAPPDSFLKVNKDHVGFYRVNYEPEVWHALANIMMKDHQNFNLADRAGFIDD	660
	661	AFALARAGLLKYADALNLTRYLQNETEYIPWQRAVVAVSYIGQMVEDDKALYPKFQRYFG	720
	721	SLVKPIASELKWENDEDHIKSLLRTTVLEFACNMDDPEALGNASLLFKNWTSGISLDVNL	780
	781	RLLVYRFGMQNSGDEQAWNYMFEKYRTATLAQEKEKLLYGLASVKNITLLNRFLSCIKNT	840
	841	TLIRSQDVFTVLRYISFNSYGKTMAWDWVRLNWEYLVKRYTLNDRNLGRLISRISGTFNT	900
	901	ELQLWQMENFFERYPDAGAGEASRKQALETTKSNIEWLKQYRDDVATWLENSEQTNFI	960

Multiple amino acid sequence alignment of Cr-APP01 (Aminopeptidase A (APP)) identified in *Calloselasma rhodostoma* venom gland transcriptome in comparison to selected APP retrieved from UniProt KB database, Aminopeptidase, UniProt ID: T2HQN1. Abbreviation: Oo – *Ovophis okinavensis*

Accession		Amino acid sequence	
J3SEZ3 PDE Ca	1	MIQQKVLFISLVAVTLGLGLGLGLKESVQPQVSCRYRCNETFSKMASGCSCDDKCTERQA	60
	61	CCSDYEDTCVLPTQSWSCSKLRCGEKRIANVLCSCSDDCLEKKDCCTDYKSICKGETSWL	120
	121	KDKCASSGATQCPAGFEQSPLILFSMDGFRAGYLENWDSLMPNINKLKTCGTHAKYMRAV	180
	181	YPTKTFVNHYTIATGLYPESHGIIDNNIYDVNLNLNFSLSSSTARNPAWWGGQPIWHTAT	240
	241	YQGLKAATYFWPGSEVKINGSYPTIFKNYNKSIPFEARVTEVLKWLDLPKAKRPDFLTLY	300
	301	IEEPDTTGHKYGPVSGEIIKALQMADRTLGMLMEGLKQRNLHNCVNLILLADHGMEEISC	360
	361	DRLEYMANYFNNVDFFMYEGPAPRIRSKNVPKDFYTFDSEGIVKNLTCRKPKQYFKAYLS	420
	421	KDLPKRLHYANNIRIDKVNLMVDQQWMAVRDKKFTRCKGGTHGYDNEFKSMQAIFLAHGP	480
	481	GFNEKNEVTSFENIEVYNLMCDLLKLKPAPNNGTHGSLNHLLKNPFYTPSPAKEQSSPLS	540
	541	CPFGPVPSPDVSGCKCSSITELEKVNQRLNLNNQAKTESEAHNLPYGRPQVLQNHSKYCL	600
	601	LHQAKYISAYSQDILMPLWSSYTIYRSTSTSVPPSASDCLRLDVRIPAAQSQTCSNYQPD	660
	661	LTITPGFLYPPNFNSSNFEQYDALITSNIVPMFKGFTRLWNYFHTTLIPKYARERNGLNV	720
	721	ISGPIFDYNYDGHFDSYDTIKQHVNNTKIPIPTHYFVVLTSCENQINTPLNCLGPLKVLS	780
	781	FILPHRPDNSESCADTSPENLWVEERIQIHTARVRDVELLTGLNFYSGLKQPLPETLQLK	840
	841	TFLPIFVNPVN	900
Cr-PDE01	1	--	60
	61	--	120
	121	---------------FEQSPLILFSMDGFRAGYLEAWESLMPNINKLKICGTHAKYMRAA	180
	181	YPTKTFVNHYTIVTGLYPESHGIIDNNMYDVNLNLNFSLSSSVAKNPAWWGGQPIWNTVT	240
	241	YQGLKAATYFWPGSEVKINGSYPTIYKNYNKSMPFEARVTEVLSWLDLPKAERPDFYTLY	300
	301	IEEPDTTGHQYGPVSGQVIKALQMADQTLGMLMEGLKQRNLHNCVNLILLADHGMEEISC	360
	361	NRLEYMDSYFNKVDFFMYEGPAPRIRSKNVPKDFYTFDSEGIVKNLTCRNPKQYFKAYLS	420
	421	KDLPKRLHYVNNVRIDKVNLMVDEKWMAVRNKKFSRCKGGTHGYDNEFKSMQAIFLAHGP	480
	481	GFKGKNEVTSFENIEVYNLLCDLLKLKPAPNNGTHGSLNHLLKNPFYTPSPAKEQSSPLS	540
	541	CPFGPTPSPDVSGCKCSSITNLEKVNQRLNLNDQAKIVSEVNNLPYGRPQVLRNDNKYCL	600
	601	LHQTKYISAYSQDILMPLWSSYTIYRSQRTSVPPSALDCLRLDVRIPAVQSQICSNYQTD	660
	661	LTITPGFLYPPNFSSYNFEQYDALITSNIVPMFKGFTRLWNYFHTILLPKYAKERNGVNV	720
	721	ITGPIFDYNCDGHFDSYGTIKEHVINTKIPIPTHYFVVLTSCENQINTPLNCLGPLKVLS	780
	781	FILPHRPDNSESCADTSPENLWVEERIQIHTARVRDVELLTGLNFYSGLKQPLPETLQLK	840
	841	TFLPIFESP--	900

Multiple amino acid sequence alignment of Cr-PDE01 (Phosphodiesterase (PDE)) identified in *Calloselasma rhodostoma* venom gland transcriptome in comparison to selected PDE retrieved from UniProt KB database, Phosphodiesterase, UniProt ID: J3SEZ3. Abbreviation: Ca – *Crotalus adamanteus*

Accession		Amino acid sequence	
Q8AY45 KSPI Bc	1	MSSGGLLLLLGLLTLWAELTPVSSRKRHPDCDKPPDTKICQTVVRAFYYKPSAKRCVQFR	60
	61	YGGCNGNGNHFKSDHLCRCECLEYP	120
Cr-KUN01	1	-SSGGLLLLLGLLTLWAELTPVSSRKRHPDCDKPPDTKICQTVVRAFYYKPSAKRCVQFR	60
	61	YGGCNGNGNHFKSDHLCRCECLEYP	120

Multiple amino acid sequence alignment of Cr-KUN01 (Kunitz-type Serine Proteinase Inhibitor (KSPI))) identified in *Calloselasma rhodostoma* venom gland transcriptome in comparison to selected KSPI retrieved from UniProt KB database, Kunitz-type serine protease inhibitor, UniProt ID: Q8AY45. Abbreviation: Bc – *Bungarus candidus*
